# Antibody Heavy Chain Variable Domains of Different Germline Gene Origins Diversify through Different Paths

**DOI:** 10.3389/fimmu.2017.01433

**Published:** 2017-11-13

**Authors:** Ufuk Kirik, Helena Persson, Fredrik Levander, Lennart Greiff, Mats Ohlin

**Affiliations:** ^1^Department of Immunotechnology, Lund University, Lund, Sweden; ^2^Science for Life Laboratory, Drug Discovery and Development Platform, School of Biotechnology, KTH Royal Institute of Technology, Stockholm, Sweden; ^3^National Bioinformatics Infrastructure Sweden (NBIS), Science for Life Laboratory, Department of Immunotechnology, Lund University, Lund, Sweden; ^4^Department of Clinical Sciences, Lund University, Lund, Sweden; ^5^Department of Otorhinolaryngology, Head and Neck Surgery, Skåne University Hospital, Lund, Sweden; ^6^Science for Life Laboratory, Drug Discovery and Development Platform, Human Antibody Therapeutics, Lund University, Lund, Sweden; ^7^U-READ, Lund School of Technology, Lund University, Lund, Sweden

**Keywords:** antibody germline gene, antibody sequence, somatic hypermutation, immunoglobulin, insertion and deletion, substitution

## Abstract

B cells produce antibodies, key effector molecules in health and disease. They mature their properties, including their affinity for antigen, through hypermutation events; processes that involve, e.g., base substitution, codon insertion and deletion, often in association with an isotype switch. Investigations of antibody evolution define modes whereby particular antibody responses are able to form, and such studies provide insight important for instance for development of efficient vaccines. Antibody evolution is also used *in vitro* for the design of antibodies with improved properties. To better understand the basic concepts of antibody evolution, we analyzed the mutational paths, both in terms of amino acid substitution and insertions and deletions, taken by antibodies of the IgG isotype. The analysis focused on the evolution of the heavy chain variable domain of sets of antibodies, each with an origin in 1 of 11 different germline genes representing six human heavy chain germline gene subgroups. Investigated genes were isolated from cells of human bone marrow, a major site of antibody production, and characterized by next-generation sequencing and an in-house bioinformatics pipeline. Apart from substitutions within the complementarity determining regions, multiple framework residues including those in protein cores were targets of extensive diversification. Diversity, both in terms of substitutions, and insertions and deletions, in antibodies is focused to different positions in the sequence in a germline gene-unique manner. Altogether, our findings create a framework for understanding patterns of evolution of antibodies from defined germline genes.

## Introduction

Antibodies, central components of humoral immunity, are crucial to our survival. The immune system allows antibodies to evolve in efforts to enhance their ability to mediate protection against disease. The biochemistry and mechanism of this complex evolution process has been extensively studied at the molecular level (1). Technological advances in sequencing and single cell analysis technology have recently allowed us to, at great depth, study antibody sequences as they develop *in vivo* (2). Indeed, various sequencing strategies and bioinformatics pipelines have been generated to allow such analysis (3, 4).

Studies of the development of humoral immune responses require knowledge of the repertoire of genes that are available in the genome. Such information allows us to properly analyze germline gene rearrangement events and hypermutation, as exemplified by extensive studies of the response against the envelope protein of HIV-1 (5, 6). Databases and associated analysis tools, like IMGT/IMGT V-QUEST/IMGT HighV-QUEST (7), have consequently been built to allow efficient analysis of antibody-encoding sequences, their genetic origin, and their evolution. Common concepts, like standardized framework and complementarity determining regions (FR and CDR, respectively), the latter of which is considered to represent the antigen-contacting part of the antibody, are commonly used in such analysis. However, numerous definitions of these regions exist in parallel (8–13), highlighting the difficulties associated with the establishment of a clear-cut definition of these regions. We hypothesized that a thorough understanding of the ways through which antibodies derived from different germline genes evolve as a consequence of somatic mutation processes will aid the establishment of such definitions. Such understanding will also aid a proper mutational analysis of clones that populate immune responses.

In this study, we have focused our attention to human IgG encoded by unsorted cells in bone marrow (BM) (14), a major site of antibody production, to define how evolution proceeds in antibody heavy (H) chains derived from 11 commonly used germline genes. The advent of high throughput next-generation sequencing methodology, and its application to studies of antibody gene sequences (2), allowed us to decipher the mutability of antibodies of different origins in ways not possible in the recent past. The analysis was highly enhanced by germline gene inference technology (15) that defined the germline gene/allele repertoires of the donors under study, thereby minimizing errors originating from inappropriate gene assignment. We now demonstrate how antibodies of different germline gene origins evolve residues and introduce insertions and deletions into CDRs and FRs. This information has implications for our understanding and interpretation of human immune responses.

## Materials and Methods

### Antibody-Encoding Transcriptomes

Antibody-encoding transcripts were isolated from unsorted cells of BM of six subjects diagnosed with allergic rhinitis, examined out of season of most seasonal pollen allergens (14). Transcripts encoding H chain variable (V) domains of different antibody isotypes were individually amplified by PCR, barcoded, and sequenced using Illumina MiSeq technology (14). Sequences are available from the European Nucleotide Archive accession number PRJEB18926. Reads were processed by pRESTO (16) and transcripts encoding each isotype were analyzed by IMGT HighV-QUEST (17) as previously described (14). A summary of the number of sequences at different stages of the analysis pipeline is provided in Supplementary Table EIV in Levin et al. (14).

### Germline Gene Repertoire

The germline gene repertoire of the donors have been inferred using IgDiscover (15) using the IgM-encoding transcriptomes of the donors’ BM, and has, when possible, been quality controlled by haplotype-based analysis (18, 19). Eleven commonly expressed germline genes (IGHV1-8, IGHV1-18, IGHV2-5, IGHV3-7, IGHV3-11, IGHV3-21, IGHV3-23, IGHV4-39, IGHV4-59, IGHV5-51, and IGHV6-1) (Table 1 in Supplementary Material) mostly encoded by a single or a few highly related alleles, representing six germline gene subgroups, were selected for further analysis (Table 1). Full-length (all codons from 1 to 105) sequences of functional germline genes were downloaded from the IMGT database^1^ (release 201718-0). Sequence similarity between these genes/alleles was determined after alignment using the ClustalW algorithm (20) as implemented in MacVector 15.5.0 (MacVector, Inc., Apex, NC, USA). Hot-spots for mutation of individual germline genes/alleles were identified by analysis through use of IMGT V-QUEST (21).

**Table 1:** Examples of germline gene allele repertoire of the six lymphocyte donors as assessed using the IgM-encoding transcriptome.

Germline	Allele composition of each donor^a^					
	Donor 1	Donor 2	Donor 3	Donor 4	Donor 5	Donor 6
IGHV1-2^b^	*02, *p06	*02, *04	*02	*02	*04, *p06	*02, *p06
IGHV1-8	*01	*01	*01	*01	*01	*01
IGHV1-18	*01	*01	*01	*01	*01	*01
IGHV2-5	*02	*01, *02	*02	*01, *02	*02	*01, *02
IGHV3-7^c^	*01, *02	*01	*01 *02	*01, *02	*01, *03	*01
IGHV3-11	*01, *03	*01	*01, *03	*01	*01, *06	*01
IGHV3-21	*01	*01	*01	*01	*01	*01
IGHV3-23	*01	*01	*01	*01	*01	*01
IGHV4-39^d^	*01	*01	*01 *07	*01	*07	*01
IGHV4-59^e^	*01, *08	*01	*01, *08	*01	*01	*01, *08
IGHV5-51	*01	*01	*01	*01	*01	*01
IGHV6-1	*01	*01	*01	*01	*01	*01

*Sequence sets not used in the analysis due to an inability to precisely define the germline origin of each protein sequence in at least three donors are showed with a gray background*.

*^a^Several alleles with identical nucleotide sequence in the assessed parts of the gene may exist, in which case only the lowest allele number is shown*.

^b^IGHV1-2*p06 is a sequence variant (*T*163*C*) of IGHV1-2*02 that is not present in the IMGT germline gene database.

^c^There is no difference in amino acid sequence in the analyzed part of the sequence of IGHV3-7*01, IGHV3-7*02, and IGHV3-7*03.

^d^There is no difference in amino acid sequence in the analyzed part of the sequence of IGHV4-39*01 and IGHV4-39*07.

^e^There is no difference in amino acid sequence the analyzed part of the sequence of IGHV4-59*01 and IGHV4-59*08.

### Analysis of Diversification of Residues Encoded Proteins

Data defining productive sequences with an origin in investigated germline genes were retrieved following IMGT HighV-QUEST-based analysis (17). Only sequences not showing evidence of insertions and deletions were scored with respect to presence of substitutions. The frequency of each amino acid was calculated for each position within the range of codons from 27 to 104. Such analysis was performed only for donors that were homozygous for a given allele or heterozygous for alleles that encode identical protein products from the analyzed part of their unmutated sequence. Sequence variability was calculated as the number of amino acids encoded by more than 1% of all reads, divided by the fraction of reads encoding the most common residue. For comparison with real protein structures, examples of structures with an origin in IGHV1-18 (PDB: 3SDY) (22), IGHV1-8 (PDB 3X3G and 3U1S) (23, 24), IGHV2-5 (PDB: 3QRG), IGHV3 subgroup (PDB: 2R56 and 3FZU) (25, 26), IGHV4-39 (PDB: 5C6T) (27), IGHV4-59 (PDB: 3HI1) (28), and IGHV5-51 (PDB: 4BUH) (29) were identified using the IMGT/3Dstructure-DB web interface (30), and coordinates were downloaded from RCSB Protein Data Bank.^2^ Structures were visualized using MacPyMOL v1.8.0.6. Sequence numbering and CDR and FR definitions are those defined by the IMGT nomenclature (13).

### Insertions and Deletions

Somatic hypermutation not only involves base substitutions but also insertions and deletions in the coding sequence (31, 32). The positions of such productive (in-frame) modifications were scored in each read based on IMGT HighV-QUEST analysis (17).

### Evidence of Selection

The 10 most highly expressed rearrangements (based on a defined CDRH3-encoding sequence) of six germline genes, IGHV1-18, IGHV2-5, IGHV3-23, IGHV4-39, IGHV5-51, and IGHV6-1, were investigated. Sequences (codons 27–104) were only retrieved from donors homozygous for a given allele to eliminate the risk of incorrect allele assignment that would contribute to perceived sequence diversification. The sequence with the highest number of counts was chosen so as to minimize the impact of random errors introduced by PCR and/or sequencing artifacts. Sequences showing evidence of insertion or deletion were not used, as the analysis pipeline is incompatible with such modes of antibody diversification. The resulting sequences of IGHV1-18 (*n* = 60 sequences), IGHV2-5 (*n* = 30 sequences), IGHV3-23 (*n* = 60 sequences), IGHV4-39 (*n* = 50 sequences), IGHV5-51 (*n* = 60 sequences), and IGHV6-1 (*n* = 60 sequences) were analyzed for evidence of positive and negative selection using Bayesian Estimation of Antigen-Driven Selection in Immunoglobulin Sequences (BASELINe, version 1.3) using a web-based interface.^3^ Focused selection statistics and the Human S5F somatic hypermutation targeting model were used for this assessment (33, 34).

## Results

### Individual Germline Repertoires

Bone marrow had been obtained from six individuals with different germline gene repertoires, a material that has previously been used for assessment of antibody repertoires in allergic subjects out of season of exposure to most environmental allergens (14). This dataset was now reanalyzed to assess antibody diversification. In any such material, allelic diversity will contribute to antibody diversity and will compromise computational analysis of antibody evolution unless correct allele assignment is made. In particular, as differences between alleles often are small, incorrect allele assignment of hypermutated genes cannot be avoided. Prior analysis of these donors’ IgM repertoires, repertoires that carry large numbers of unmutated sequences, can be used to ensure proper downstream analysis of IgG repertoires. Such analysis (19) was performed using IgDiscover (15) to define the lymphocyte donors’ IGHV germline gene and allele makeup. We furthermore used a haplotype quality-control approach to define the validity of many of the allele calls (18, 19). This approach also allowed us to validate novel alleles not present in the IMGT reference directory used for gene assignments (19). By using donors with defined germlines, we minimized the risk of introducing artifacts in our analysis of the mutational paths taken by antibodies of different germline gene origins. Importantly, this approach identified allele IGHV1-2*p06 (IGHV1-2*02 T163C) in three of six individuals (19) (Table 1), an allele that is not identified by standard IMGT HighV-QUEST or V-QUEST analysis. A failure to identify this allele would incorrectly have enhanced the perceived substitution frequency in one position of this gene by the approach taken in this study. As a consequence of the substantial, but difficult to detect, allelic diversity of IGHV1-2, it was not included in this study. We also made sure that the investigated germline genes were not extensively similar to alleles of other germline genes in the donors’ repertoires, as such similarity may, following hypermutation, incorrectly relate products of other genes to the genes under investigation. IGHV3-23 and IGHV3-23D are in this context treated as one gene as they are identical in sequence. Among the other genes, only one investigated allele of IGHV4-59 had a highly similar allele defined by IMGT (>98% nucleotide identity) assigned to another gene location (Figure S1 in Supplementary Material), but this other allele (IGHV4-4*08) was not present in the repertoires of the investigated individuals.

**Figure 1:**
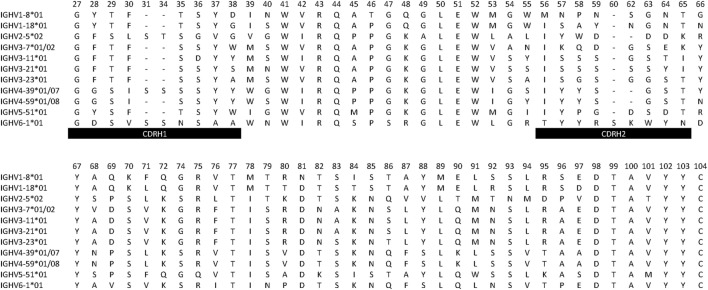
Protein sequences (residues 27–104) encoded by germline genes primarily investigated in this study. Boundaries for complementarity determining regions (CDRs) and residue numbering are as defined by the IMGT nomenclature (13).

Overall, several germline genes were highly diverse (different alleles used by different donors or presence of different alleles in a given individual), but others were not, some of which were used for this study (Table 1). In all, our analysis focused on a set of commonly used “core” genes (35) utilized in rearrangements obtained from individuals conceived to be homozygous for a given allele or heterozygous for alleles expected to encode identical protein sequences in their unmutated form. As the analysis focused on sequences (germline-encoded protein sequences are shown in Figure 1) from CDRH1 up to the end of FR3, alleles that encode identical protein products in this part of the H chain variable domain could be included in the study. Thus, for the purpose of analysis of selection associated with hypermutation, only sequences derived from individuals homozygous for a given gene sequence encoding CDRH1 to FR3 (codons 27–104) were used.

### The IgG Population Encoded by BM Is Highly Somatically Evolved

We chose to study mutations in antibodies of the IgG isotype produced in BM as this is a major site of antibody production. By focusing on the entire transcriptome and not sequences collapsed to individual clones, the analysis also focused on features of highly produced products. Samples (10 ml) obtained from BM, as analyzed in this study, are reproducible representations of antibodies produced at this site (14). This population of cells largely contains mutated transcripts as evidenced by the fact that only 1.4% (range 0.6–2.5%) of them showed a level of mutation below 2% at the nucleotide level (the corresponding IgM-encoding transcriptome contained 45% (range 35–55%) of sequences displaying a degree of mutation below 2%) (14), as determined using IMGT HighV-QUEST.

### Germline Genes Differ Extensively in the Extent of Targeting by Substitutions

**Figure 2:**
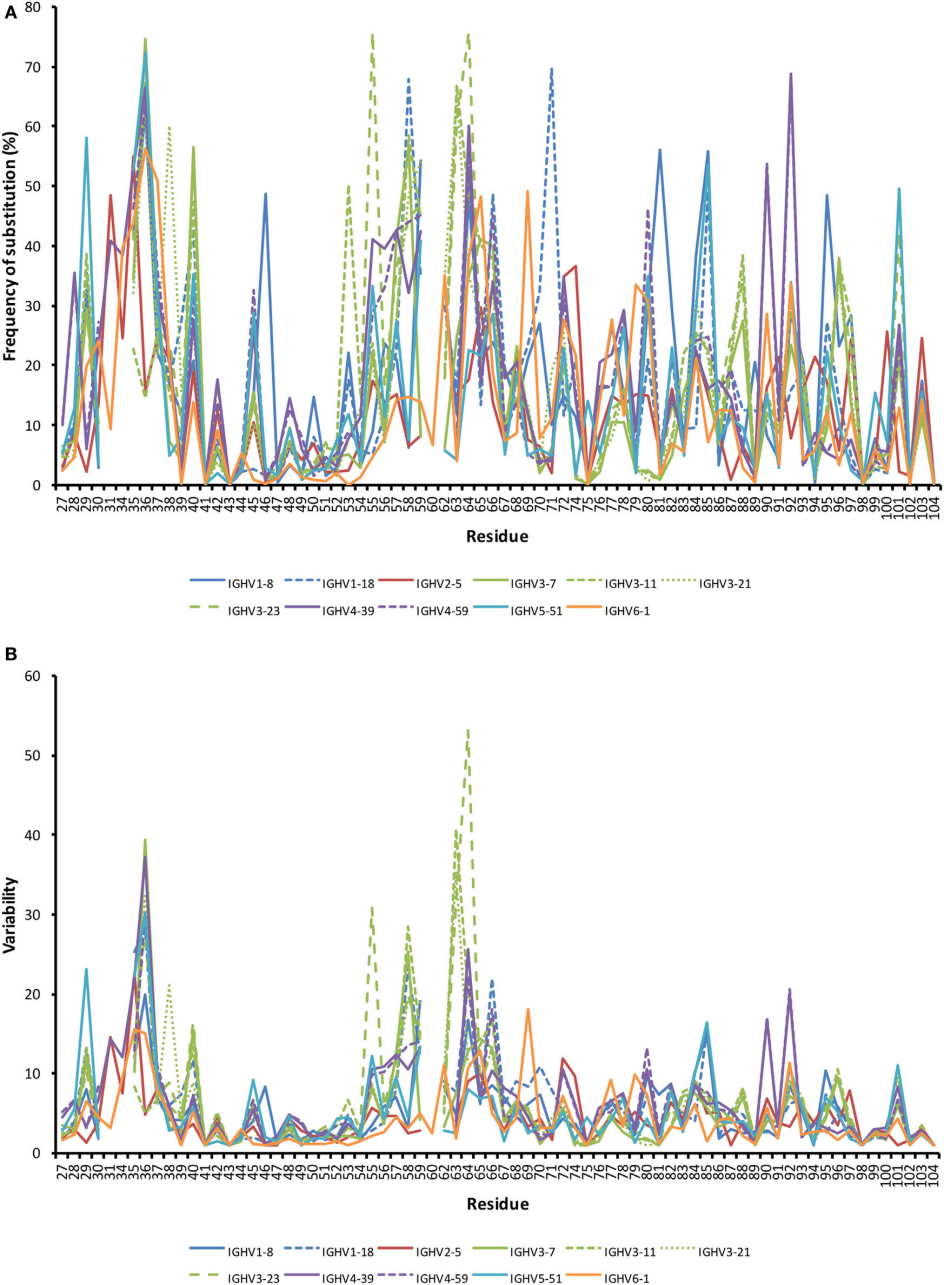
Frequency of substitutions **(A)** and degree of variability **(B)** of residues encoded by transcripts in bone marrow (BM) with an origin in the 11 investigated germline genes. Residues 27–38 code for complementarity determining region (CDR) 1, while residues 56–65 code for CDR2. Variability was calculated as (the number of amino acids encoded by more than 1% of all reads)/(fraction of reads encoding the most common residue). Only sequences showing no evidence of insertions and deletions were included in the analysis. Numerous residues differed substantially between germline genes in the degree of targeting by substitution. Note the substantial degree of substitution also in some residues of framework region (FR). Frequency of substitution and variability for individual genes are shown in Figure S2 in Supplementary Material.

The degree of substitution in the part of VH encoding CDR1, FR2, CDR2, and FR3 with an origin in 11 well-defined germline genes from six human IGHV germline gene subgroups was analyzed. The average frequency of substitution of a residue from residues 27 to 104 ranged from 12.5% (IGHV2-5) to 18.4% (IGHV4-39). Different substitution patterns were seen with different residues being targeted by diversification depending on germline gene origin (Figure 2). Substitutions were, as expected, often located to some residues within CDRs, but they also occurred frequently in numerous residues in FRs. It was not uncommon for FR residues encoded by a particular germline gene to be substituted in >25% of all transcripts. Diversification of FRs is thus an important aspect of antibodies produced from cells in BM that have undergone a somatic hypermutation processes.

**Figure 3:**
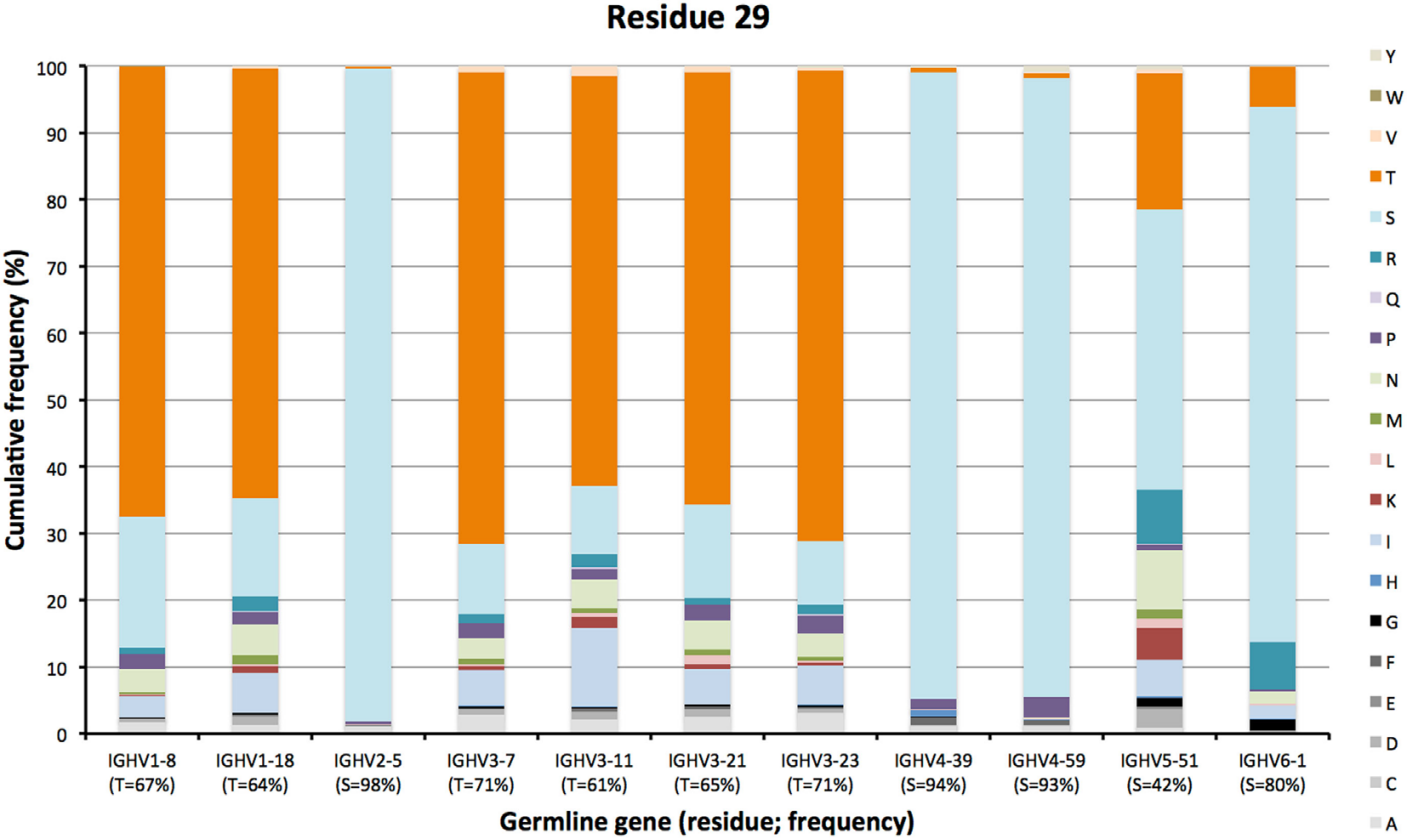
Diversity of residues encoded by transcripts of different germline gene origins in position 29 of VH of IgG. The germline-encoded residues and their frequencies in IgG-encoding transcripts are shown at the bottom of the graph. Note the extensive diversification of S29 in IgG-encoding transcripts derived from IGHV5-51 but not from other genes, in particular IGHV2-5, IGHV4-39, and IGHV4-59.

**Figure 4:**
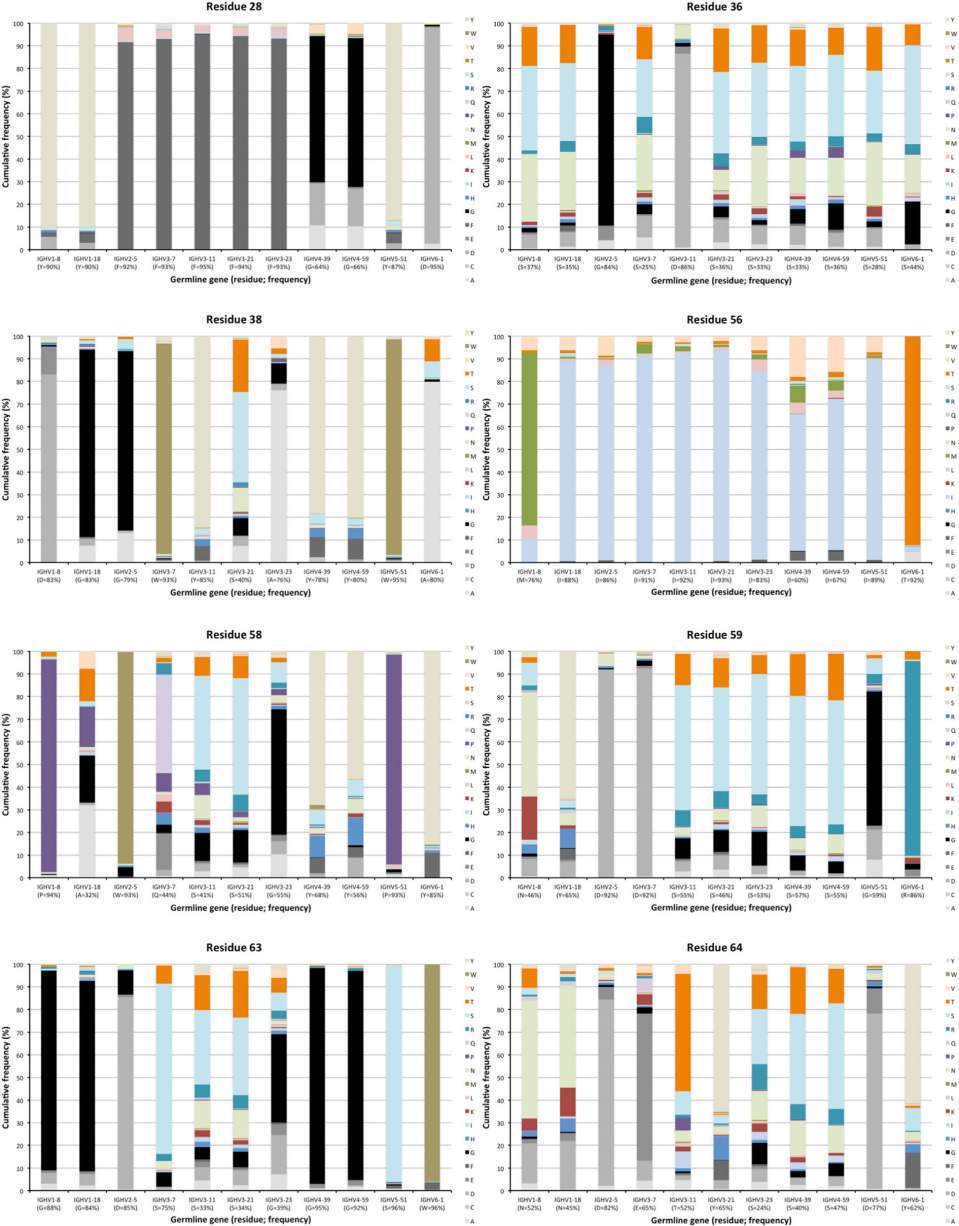
Examples of differences in diversification of residues in complementarity determining region (CDR) encoded by transcripts of different germline gene origins. Residues shown include positions 28, 36, and 38 of CDRH1, and positions 56, 58, 59, 63, and 64 of CDRH2 of IgG. The germline-encoded residues and their frequencies in IgG-encoding transcripts are shown at the bottom of the graph. Substitutions are introduced in 5–36% (residue 28), 14–75% (residue 36), 5–60% (residue 38), 7–40% (residue 56), 6–68% (residue 58), 8–54% (residue 59), 4–67% (residue 63), and 18–76% (residue 64) of the transcripts depending on their different germline gene origins.

**Figure 5:**
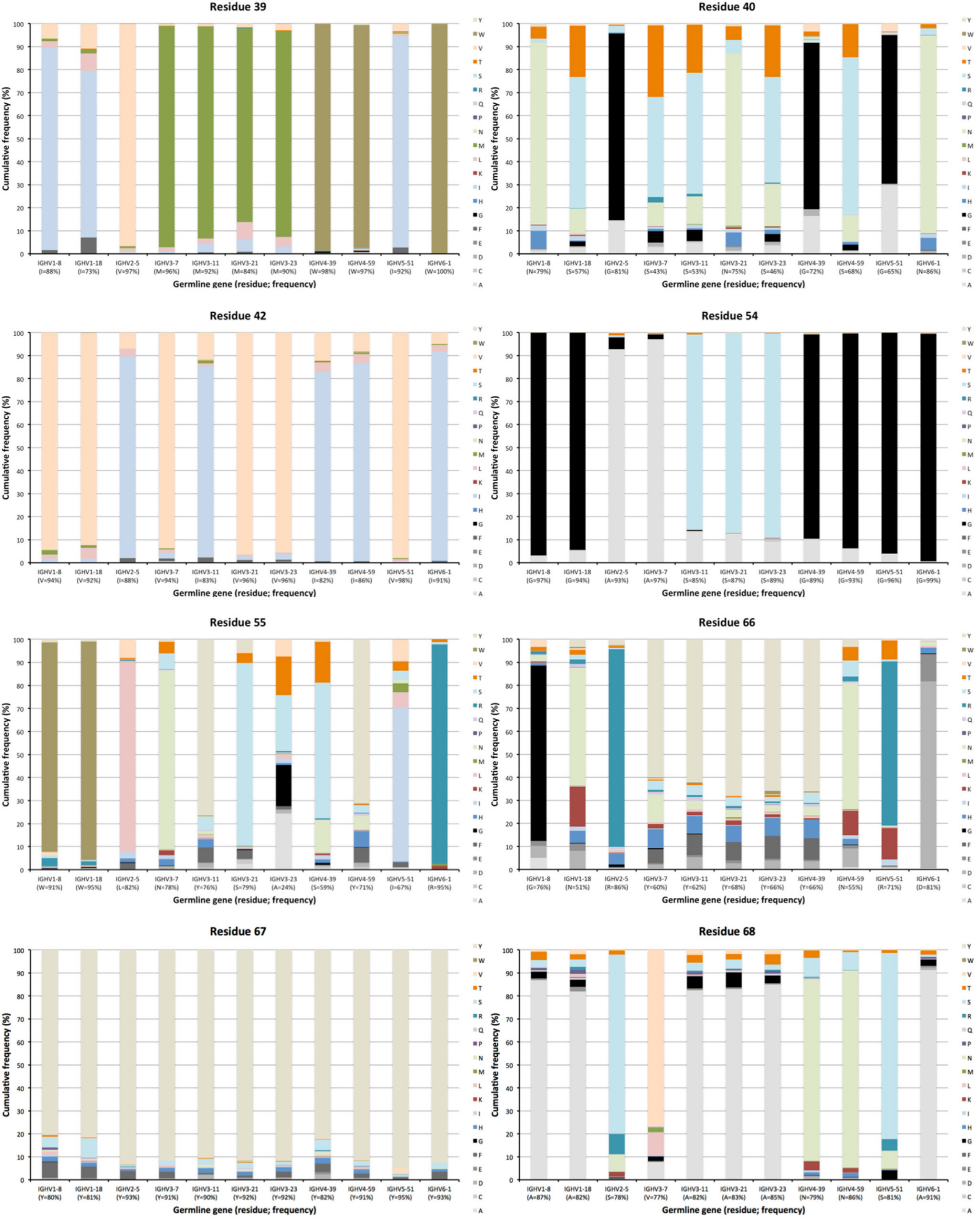

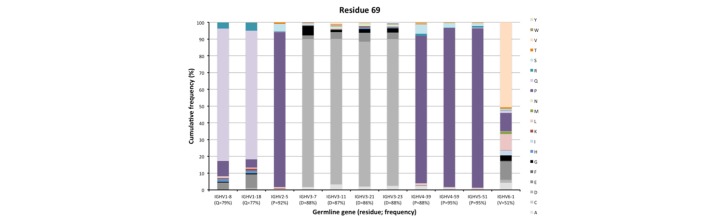
Extent of diversification of residues close to complementarity determining region (CDR) in the linear protein sequence as encoded by transcripts of different germline gene origins. Residues shown include residues 39, 40, 42 immediately after CDRH1, and residues 54–55 immediately before and residues 66–69 immediately after CDRH2 of IgG. Residue 41, a conserved tryptophane belonging to the domain’s core, is not diversified in products encoded by any germline gene. The germline-encoded residues and their frequencies in IgG-encoding transcripts are shown at the bottom of the graph.

The degree of targeting of CDR1 and CDR2 differed substantially between different germline genes both in terms of the frequency of substitution and the degree of variability introduced (Figure 2; Figure S2 in Supplementary Material). For instance, substitutions were most frequently incorporated into CDRH2 of VH with an origin in IGHV3-11, while substitutions were incorporated more frequently into CDRH1 of VH with an origin in IGHV5-51 (Figure S2 in Supplementary Material). The precise codons targeted by successful diversification differed between germline genes. For instance, while residue 29 (mostly S or T in germline sequences) was targeted by substantial diversification in proteins derived from some genes (e.g., investigated genes of subgroups 1, 3, and 5) it was not targeted in genes of other germline origins (IGHV2-5, IGHV4-39, IGHV4-59, and IGHV6-1) (Figure 3) (additional examples are provided in Figure 4). In the case of S29-encoding germline genes, the extensive targeting of mutations to this residue in IGHV5-51 was associated with the presence of a mutational hotspot in the codon of this gene (Figure S3 in Supplementary Material). Similarly, residues in immediate proximity to CDR in the linear sequence were frequently diversified. The extent of diversification of some of these residues differed substantially depending on germline gene origin. For instance, while W55 of IGHV1-8 and IGHV1-18 and R55 of IGHV6-1 were rarely (<10%) substituted, A55 of IGHV3-23 was substituted in products encoded by 76% of the transcripts with an origin in this germline gene (Figure 5). Among these genes, IGHV6-1 carries an AA dinucleotide hotspot motif while IGHV3-23 carries a TA motif and a AGCT motif that may specifically target codon 55 with mutations.

In summary, VH domains encoded by transcriptomes found at a major site of antibody production, the BM, differ in the paths through which they evolve residues within or in the immediate vicinity to CDRs.

### Evolution of Residues Belonging to the Cores of Variable Domains

**Figure 6:**
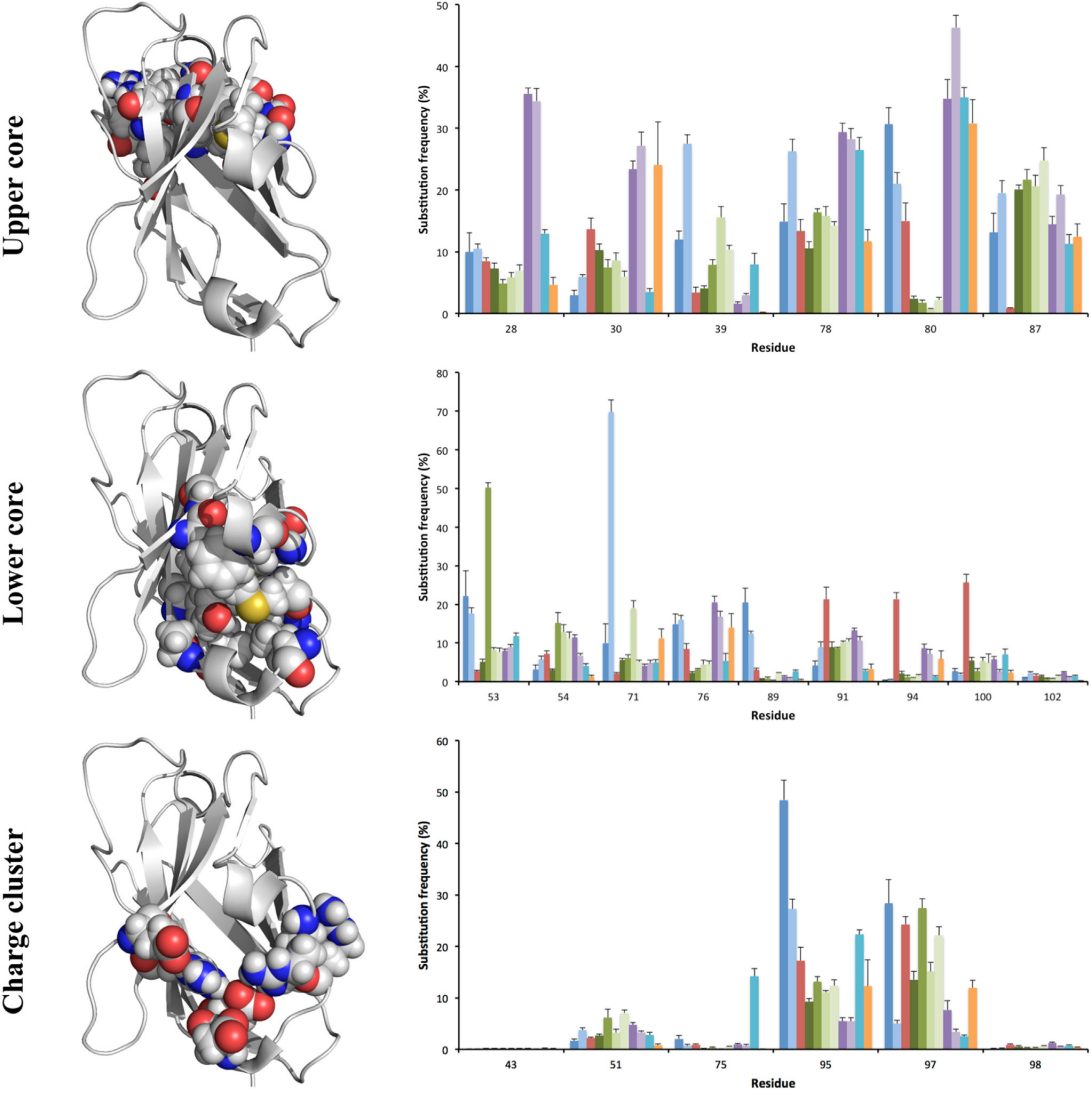

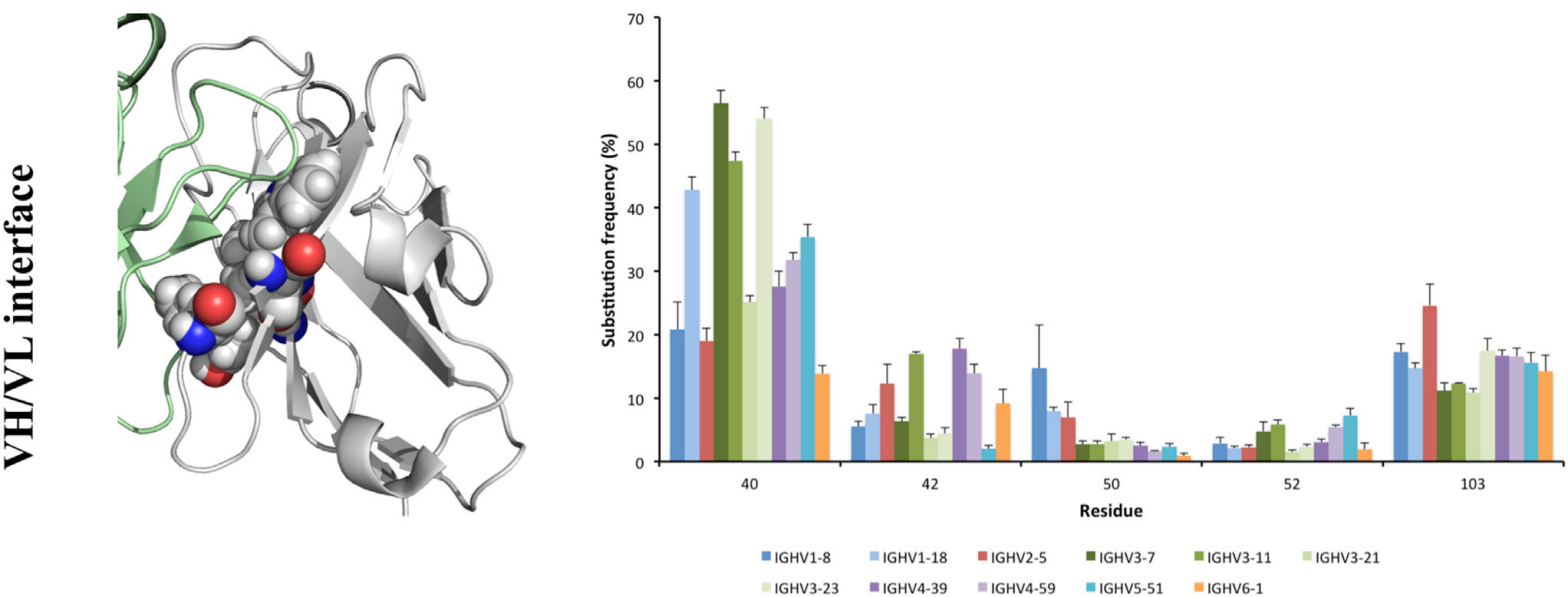
Substitution frequency of the charge cluster, lower and upper cores, and major residues that participate in VH/VL interface of the antibody H chain. Error bars indicate SEM between repertoires of different donors. Residues belonging to these structures include those exemplified in the structure of the VH of a human antibody fragment with an origin in germline gene IGHV1-18 (PDB: 3SDY). The VH domain is shown with a gray backbone. Part of the VL domain in the illustration of the domain interface is shown in green. Achieved diversity of individual residues in the sequence populations are outlined in Figures 7–9, and Figure S4 in Supplementary Material.

Residues that make up the core regions of antibodies are important for protein stability, and may thus conceivably be less targeted by mutation. Indeed, five residues within the region CDRH1-FR3 of VH were substituted in <2.5% of all human IgG transcripts independently of their germline gene origin. These included W41 and C104 in the domain’s central core, residues R43 and D98 in the domain’s charge cluster, and residue Y102 in the lower core of the domain (Figure 6). Nevertheless, as described below, we observed several other residues belonging to the core regions that are diversified more extensively during somatic antibody evolution.

**Figure 7:**
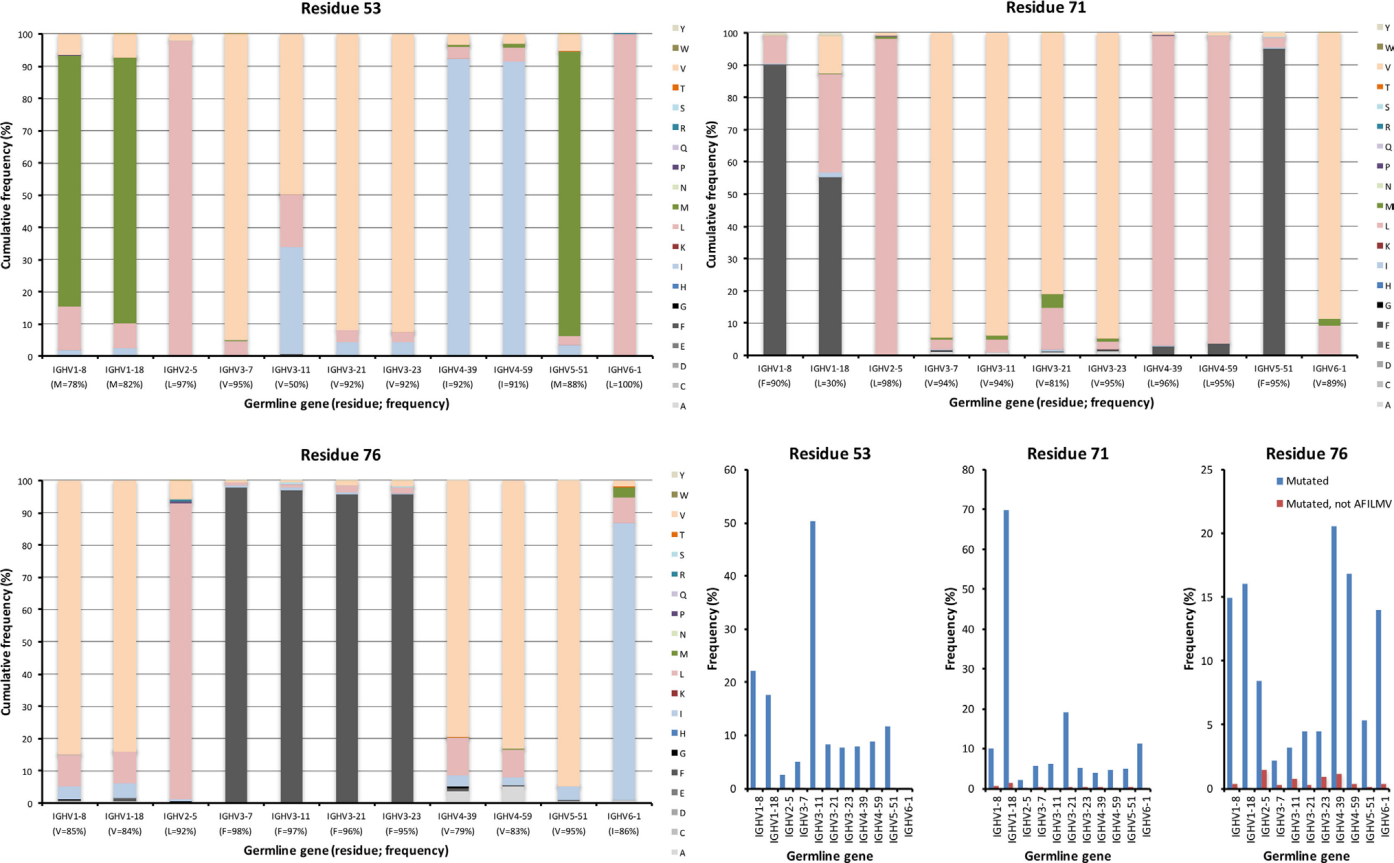
Substitutions of lower core residues 53, 71, and 76 as introduced during somatic hypermutation processes of IgG-encoding genes derived from different germline genes. The diversification of these mostly hydrophobic residues largely results in introduction of other hydrophobic residues (panels to the bottom right).

The lower core is shielded from the upper core by the highly conserved central core (including, e.g., W41 and C104) and a more direct influence by substitutions in this core on the binding site may be limited. Instead, lower core residues may affect the biophysical properties of the domain (36). Several of these residues (of which residues 53, 54, 71, 76, 89, 91, 94, 100, and 102 were assessed in this study) were essentially untouched by somatic diversification, while others, depending on germline gene origin, were diversified. In particular, residue 53 of IGHV3-11 (but not IGHV3-7, IGHV3-21, and IGHV3-23) and residue 71 of IGHV1-18, were prone to diversification (Figures 6 and 7). IGHV1-18 encodes a L at position 71, while other genes of the IGHV1 subgroup encode F in this position. Mutation of this codon in IGHV1-18 introduced F in this position at a high frequency (55%). Mutation of residue 53 incorporated conserved hydrophobic substitutions in place of the germline-encoded residue. In the case of IGHV3-11, V was mainly substituted by L or I. Similarly, mutation of residue 76 largely introduced conservative, hydrophobic substitutions (Figure 7). In summary, some residues of the VH domain’s lower core are targets for conservative hypermutation.

**Figure 8:**
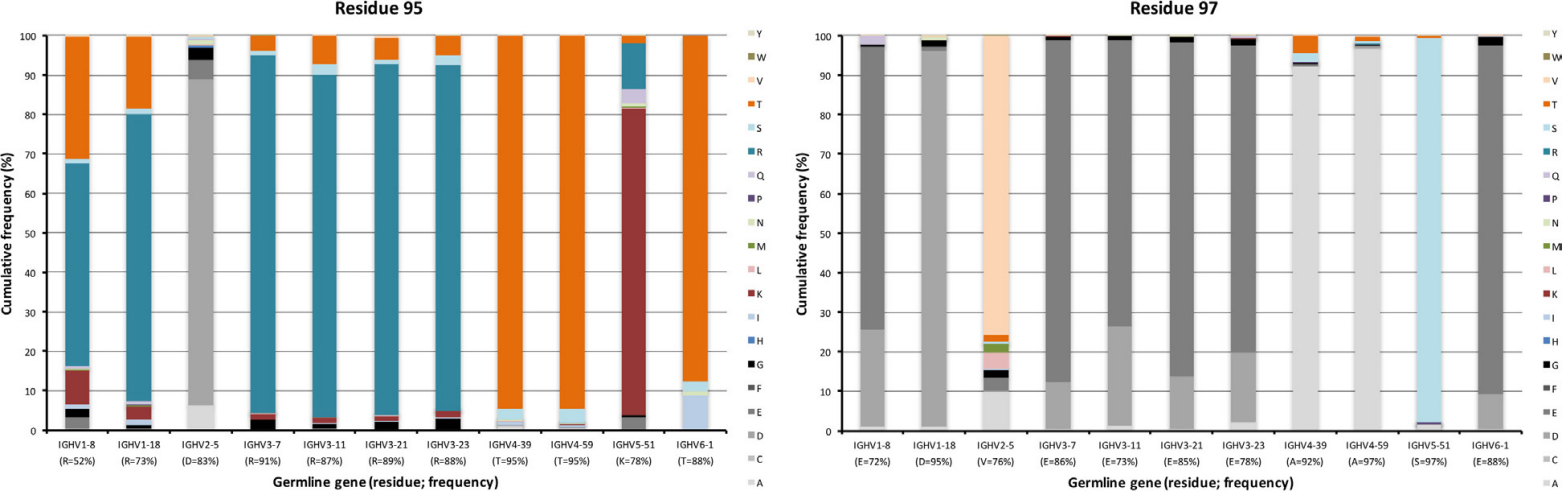
Substitutions of charge cluster residues 95 and 97 as introduced during somatic hypermutation processes of IgG-encoding genes derived from different germline genes.

A cluster of charged residues is situated close to the lower core of VH (36). It involves residues at positions 43, 51, 75, 95, 97, and 98 (37). Of these, 0–2 residues, mostly residue 95 and 97, were targeted by substitutions at frequencies above 10% (Figure 6). Of note, 48% of all sequences with an origin in IGHV1-8 were targeted by substitution at residue R95 (a codon not associated with a mutational hotspot), while only 9% of sequences with an origin in IGHV3-7 was diversified in this position. Limited diversity (T or K) dominated the diversity introduced at this position. Similarly, substitution at position 97 (a codon not associated with a mutational hotspot in any of the investigated germline genes) in IGHV3 gene subgroup members was dominated by a conservative E → D mutation, while substitution of V97 in IGHV2-5, an unusual, hydrophobic side chain in this cluster, introduced a range of modifications although mainly to A (Figure 8). Altogether, there is room for diversification in the charge cluster in a germline-directed manner, modifications that for instance may affect the biophysical properties of the domain.

Substitutions of residues that belong to the VH/VL interface may affect binding site architecture. We investigated the tendency for substitution in five residues (40, 42, 50, 52, and 103), the surfaces (Figure 6) of which are substantially buried by formation of the VH/VL dimer (11). Among these, residues 50 and 52 were rarely mutated [each below 10% of transcripts, except in the case of residue 50 of IGHV1-18 (15%)] while, in particular residue 40 (G, N, or S) but also residues 42 (I or V) and 103 (Y) were frequently substituted (Figure 6), although mostly in a restricted manner (Figure 5; Figure S4 in Supplementary Material). Residue 40 of some germline gene origins showed substantial levels of substitution (even above 50%) but the diversification was largely limited in scope (such as S → N or T, or G → A) (Figure 5). In summary, there is room for diversification of some residues often buried in the VH-VL interface, modifications that may affect the binding site or the stability of the VH-VL pair.

**Figure 9:**
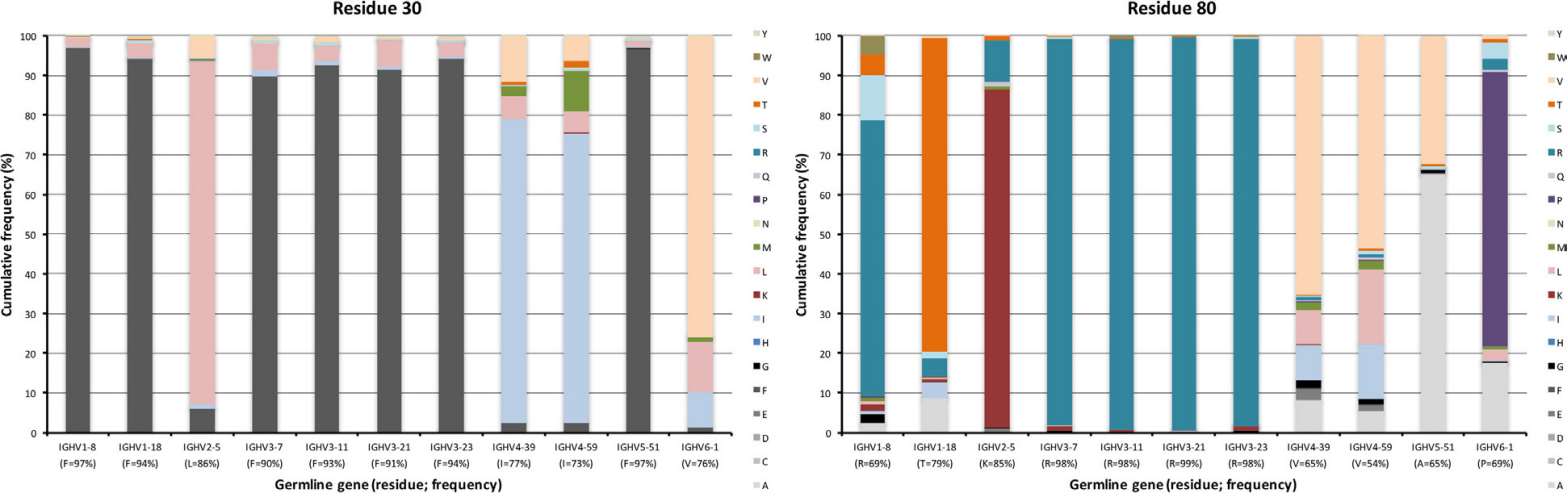
Substitutions of upper core residues 30 and 80 as introduced during somatic hypermutation processes of IgG-encoding genes derived from different germline genes. Substitution of residue 28 is shown in Figure 4.

The upper core of antibody H chain variable domains (37) (of which residues 28, 30, 39, 78, 80, and 87 have been assessed here) is located just beneath the paratope and diversification of its residues may have profound effects on the binding site (38). Several of the residues that constitute the upper core (36) are by definition part of the sequences that comprise CDR, although their side chains are not necessarily extensively exposed on the surface of the domain. The residues play different roles, depending on their biophysical nature (37). Several residues in this core of VH, depending on its germline gene origin, are prone to accept mutations. Many germline genes encode large aromatic residues at position 28 that were rarely mutated (mostly <10%) (Figures 2 and 4). However, germline genes IGHV4-39 and IGHV4-59 encode a G in this position, a residue that was frequently (approximately 35%) substituted (mostly to A, D, and V) in products of IgG-encoding transcripts. Similarly, genes that encode an aromatic side chain in position 30 of VH rarely substituted it (≤10%) while genes derived from germline genes like IGHV4-39, IGHV4-59, and IGHV6-1, which encode a hydrophobic amino acid in position 30, were more prone to substitute it, mostly for another hydrophobic residue (Figure 9). Residue 80 in the upper core is important for the positioning and conformation of CDR2 (38). IGHV3 germline genes incorporate R at this position, a side chain that was only very rarely (≤2% of reads) substituted by other residues. In contrast, R80 in antibody-encoding genes derived from IGHV1-8 underwent substitution at a high frequency. This ability for diversification is not associated with the presence of a mutational hotspot (*WA*/*TW* or *RGYW*/*WRCY*) in this codon in IGHV1-8 (Figure S3 in Supplementary Material). Other germline genes encode other residues in position 80 and these may also be substituted to a substantial extent (Figure 9). Altogether, there is tolerance for diversification of many residues of the upper core in a germline-origin-dependent manner.

### Antibody Evolution Provides Diversity Beyond CDR and Domain Core Structures

**Figure 10:**
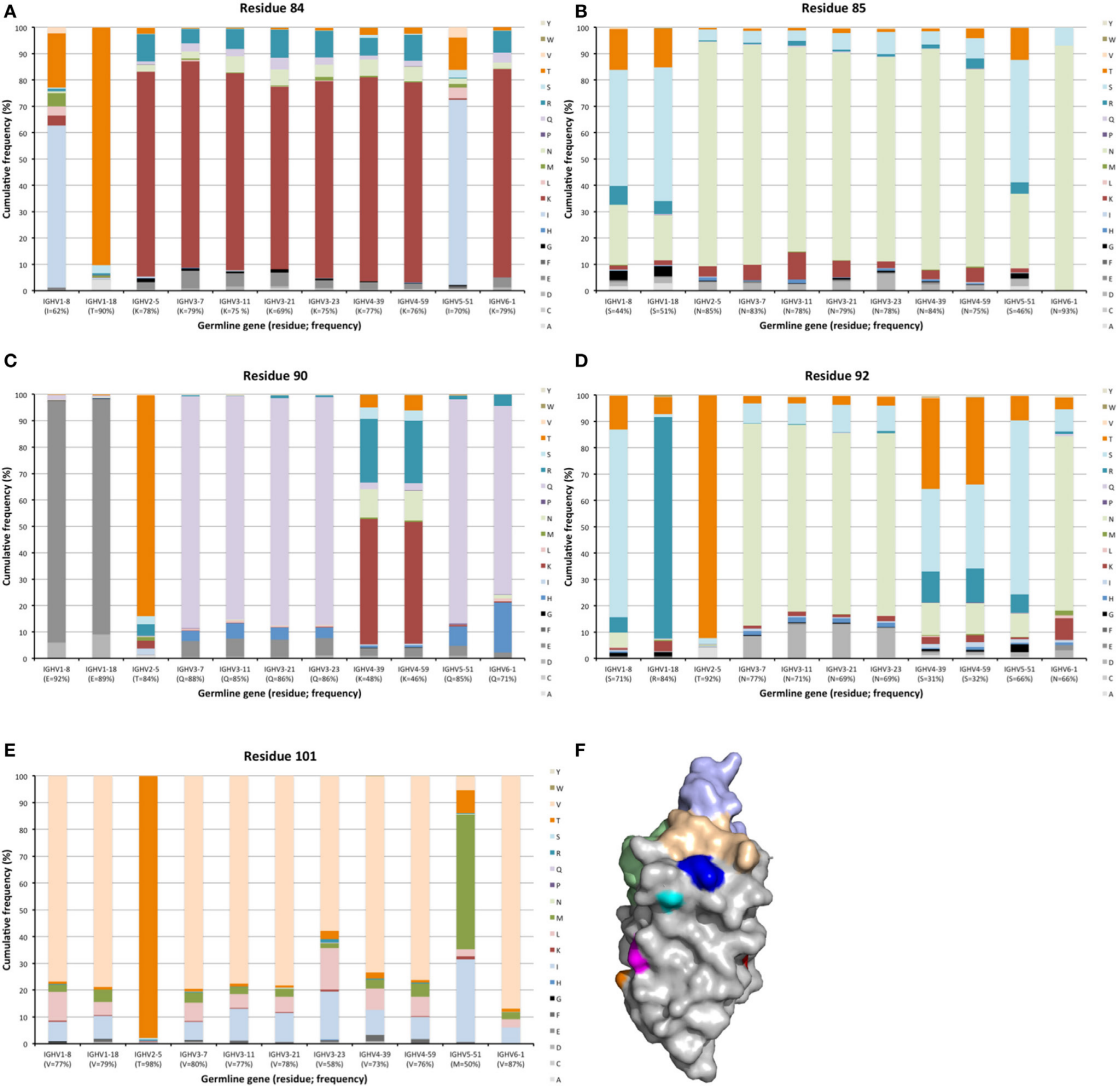
Substitutions of residues 84 **(A)**, 85 **(B)**, 90 **(C)**, 92 **(D)**, and 101 **(E)** in FR3 as introduced during somatic hypermutation processes of IgG-encoding genes derived from 11 different germline genes. Structure **(F)** of an IGHV5-51-derived scFv (PDB: 4BUH) indicating the side chain atoms of residues 84 (cyan), 85 (dark blue) in close proximity to CDR1, and of residues 90 (magenta), 92 (orange), and 101 (red). CDR are shown in brown (CDR1), green (CDR2), and light blue (CDR3).

Hypermutation may extend to surface residues beyond CDRs, even to residues that are not located in immediate proximity to those defined to make up the CDRs. Numerous residues, in particular in FR3 carried such diversity (Figures 2 and 10). Sequences around residue 85 have been considered as a fourth CDR (39). This residue frequently carried diversity, a feature particularly evident in transcripts with an origin in IGVH1-8, IGHV1-18, and IGHV5-51 germline genes, in which case about 50% of the transcripts carried substitutions. This side chain is localized immediately below CDR1 in the folded domain (Figure 10) and it is highly conceivable that mutations may affect binding affinity and/or specificity. Certainly, antibodies derived from some germline genes show extensive evolvability in this part of the domain.

**Figure 11:**
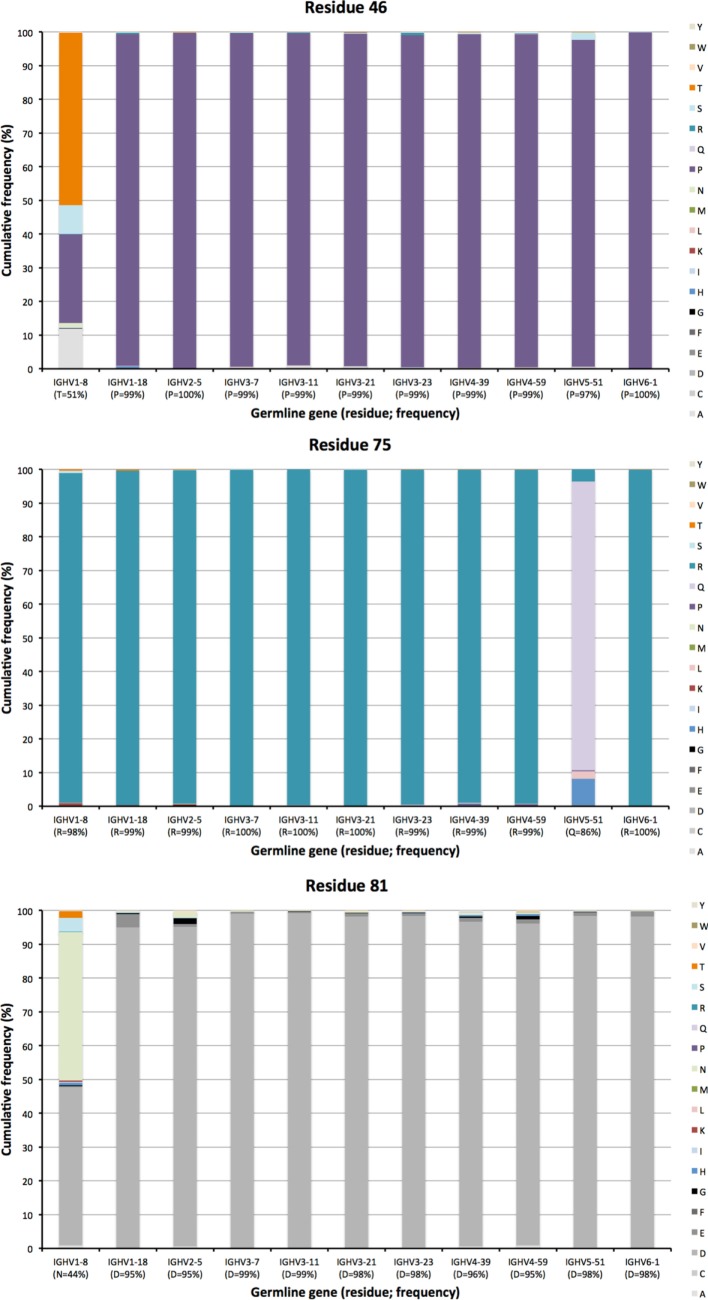
Substitutions of residues 46, 75, and 81 as introduced during somatic hypermutation processes of IgG-encoding genes derived from 11 different germline genes.

Other residues that are located at a substantial distance from CDR, were also frequently mutated. For instance, residues 90 and 92 in FR3 showed evidence of extensive diversification in transcripts derived from some germline genes, in particular those of IGHV4-39 and IGHV4-59 (Figure 10). Similarly, residue 101 in many VH carried a substantial level of diversification (Figure 10). Only in the case of IGHV3-23 was this propensity for substitution in position 101 associated with the presence of a mutational hotspot. The side chain of residue 101 is also exposed on the domain’s surface near the interface with VL far away from the binding site. Some residues, although not targeted to diversification in general, may be targeted extensively in antibodies derived from some germline genes. For instance, in similarity to residue 71 of IGHV1-18 (described above; Figure 7), residues 46 and 81 of IGHV1-8, and to some extent residue 75 of IGHV5-51 were frequently mutated (Figure 11). The corresponding codons of the germline genes encode T, N, and Q, respectively, while most other germline genes encode P, D, and R, respectively (Figure 1). The evolution of in particular IGHV1-8-derived H chain variable domain often introduced precisely these residues into the products.

In summary, numerous FR residues, the side chains of which are found on the surface of VH, are diversified in a germline gene-defined manner through antibodies’ evolution processes *in vivo*.

### Insertions and Deletions

**Figure 12:**
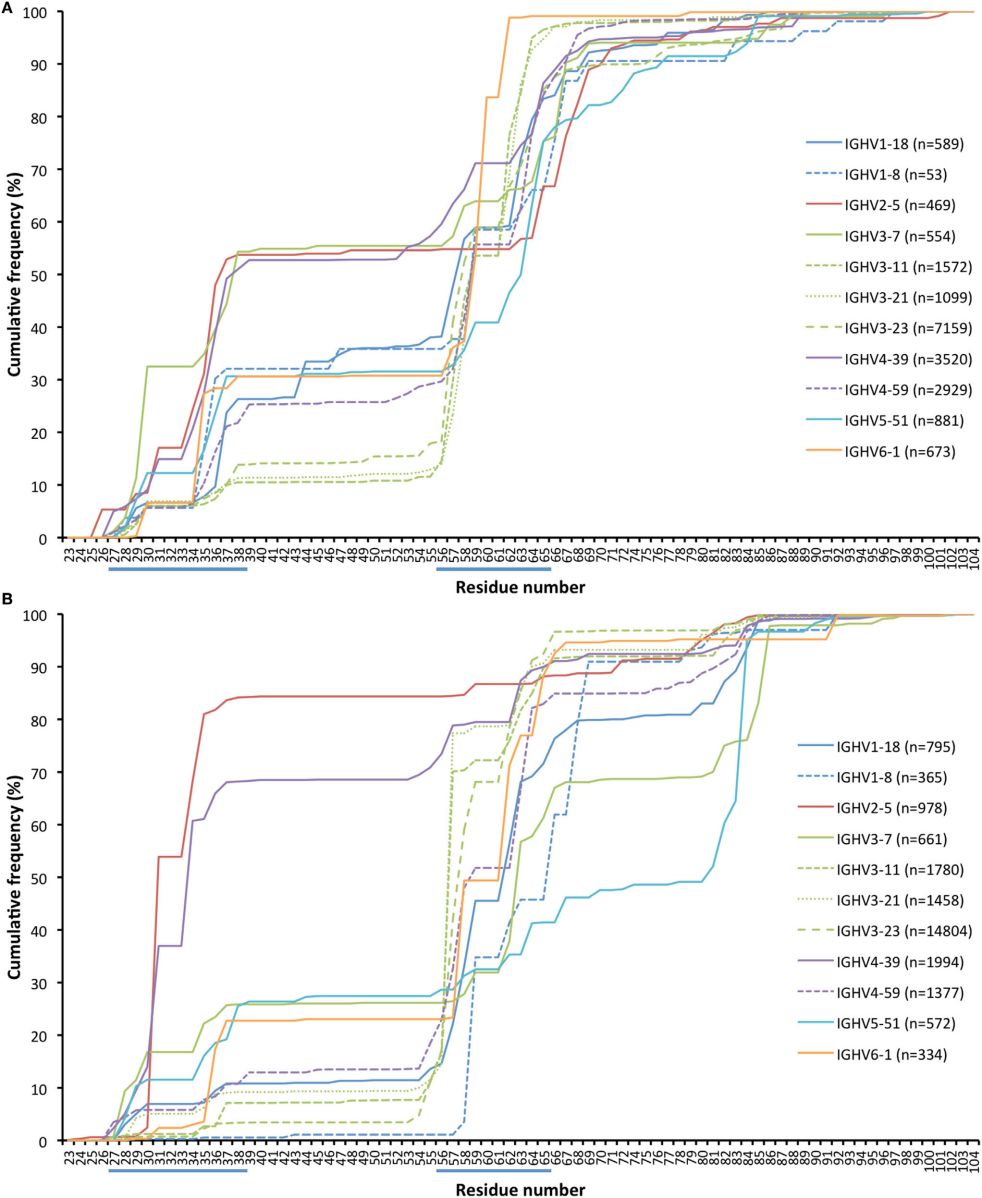
Cumulative frequency of in-frame codon insertion **(A)** and deletion **(B)** (as calculated by IMGT HighV-QUEST) in rearranged genes derived from a set of germline genes (irrespective of allele origin). The occurrence of such events in genes derived from additional germline genes, not representing the core genes investigated in this study, are shown in Figure S5 in Supplementary Material.

Antibody sequences can evolve not only by hypermutation but also by insertion and deletion of entire codons (31, 32). The present dataset allows for analysis of such processes in hypermutated antibody sequences of different germline gene origins. We identified the location of such modifications (as annotated by IMGT HighV-QUEST) in transcripts derived from a number of germline genes (Figure 12; Figure S5 in Supplementary Material). Insertions were on average longer than deletions (6.7 and 4.6 bases, respectively; *p* = 0.028 using the Wilcoxon signed rank test) in in-frame transcripts with an origin in the 11 germline genes (irrespective of allelic origin) that are the focus of this study. Most such modifications occurred within/close to CDRs. Several genes (like IGHV3-23) were primarily targeted by insertions and deletions in CDRH2 while others (like IGHV2-5 and IGHV4-39) were targeted also by such modifications in CDRH1. Some genes [like IGHV3-7, IGHV5-51 (Figure 12), and IGHV1-69 (Figure S5 in Supplementary Material)] also extensively introduced insertions and deletions in CDRH4, i.e., in the loop situated in close proximity to other, conventional CDRs. In summary, it appears that rearranged sequences derived from different germline genes target insertions and deletions to different parts of their sequence.

### Somatic Hypermutation and Evidence of Selection

**Figure 13:**
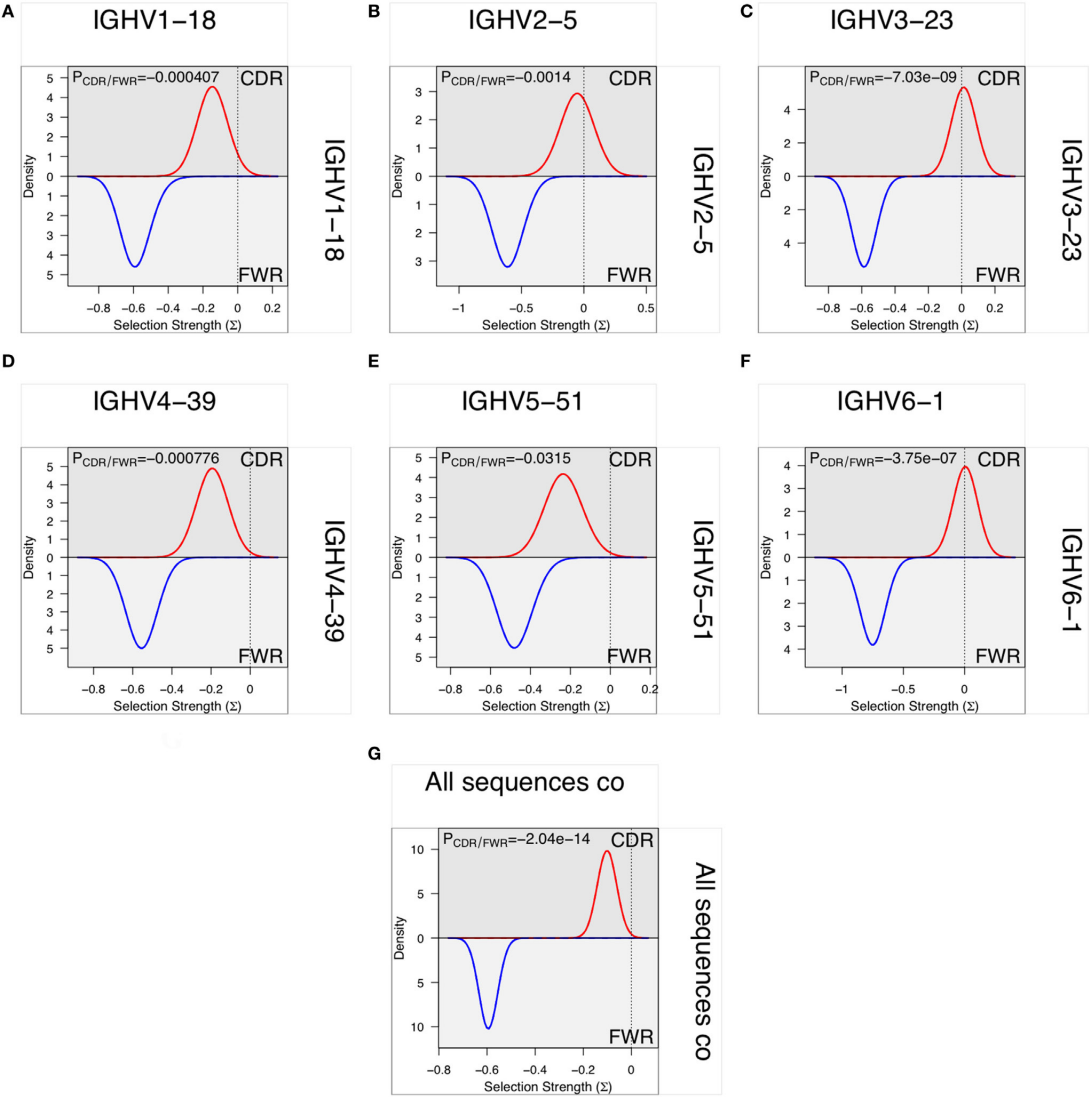
Analysis, using BASELINe (33, 34), of selection pressure on the diversification of residues of the framework region (FR) and complementarity determining region (CDR) with an origin in six germline genes [IGHV1-18 **(A)**, IGHV2-5 **(B)**, IGHV3-23 **(C)**, IGHV4-39 **(D)**, IGHV5-51 **(E)**, and IGHV6-1 **(F)**]. There is a profoundly more negative selection of diversification of residues of FR2 and FR3 than of CDR1 and CDR2 but there is no evidence for positive selection in the substitution pattern of CDR of sequences derived from any of the individual germline genes. Indeed, overall there is evidence for negative selection for substitution also in the CDR **(G)**.

*In vitro* evolution through hypermutation may contain objective evidence of selection as events likely to contribute to improved binding are favored over those with no or negative influence on antigen recognition. Such productive events are considered focused to the CDR and they may be detectable using computational approaches (33, 34, 40–42), although this possibility has also been questioned (43, 44). We investigated the evidence for such selection in only the most frequent, independent sequences of each germline in each donor to minimize the effect of random PCR and sequencing errors. Only donors that expressed a single allele of a gene were included in the analysis to minimize the risk of errors introduced by incorrect allele assignment. Such analysis demonstrated that there, despite the high degree of substitutions in FR of VH encoded by BM-derived transcripts, was a profound negative selection for mutations in FR. Although there was less selection against substitution of residues in CDR, it was not possible to identify positive selection in VH domains with an origin in any of the investigated germline genes (Figure 13).

## Discussion

Analysis of the information content of human antibody diversification holds promise for understanding antibody evolution and affinity maturation as it occurs *in vivo*, and evolution processes as used *in vitro* to develop high affinity, highly biophysically stable antibodies. The advent of next-generation sequencing and the availability of much larger collections of antibody sequences allows for a very in-depth analysis of antibody diversity. We envisaged that such analysis would also define constraints on human repertoire development as a function of antibody germline gene origin and thus enhance the way we in the future analyze events involving somatic diversification. Such studies and concomitant studies of antigen-antibody complex structure have been used to understand in detail how human humoral immune repertoires develop, or fail to develop efficiently. Large-scale studies, as recently reviewed, have addressed the evolution of antibodies against highly functional epitopes on viral antigens like the envelope protein of HIV-1 with the intention to enable design of immunogens that more efficiently induce protective immunity in vaccinated subjects (5, 6, 45). In the present study, the aim was to deconvolute antibody diversification paths, not from a global perspective but with a focus on products of individual germline genes, to enable enhanced quality of future analysis of antibody evolution. To do so, we employed sequences that encode IgG in BM (14), a major site of long-term, sustained antibody production. The sequences were derived from subjects diagnosed with seasonal allergic rhinitis but they were obtained out-of season of most seasonal allergens. We consequently consider them not to be biased by an on-going allergic immune response. In any event, we do not consider that major features of evolution of the IgG response of such subjects would be dramatically different from that of non-allergic, immunocompetent, subjects.

The present study focuses on the diversity found from CDR1 until the end of FR3 of the human H chain V domain. By focusing our attention on diversification of well-defined germline genes and alleles, our analysis is minimally confounded by differences in germline gene allelic makeup between individuals or between haplotypes of an individual. In preparation of the present study, we consequently inferred the germline VH repertoires of the lymphocyte donors (19) and analyzed genes of donors with well-established germline gene allele composition. The one exception to this rule is IGHV3-23 and its, in their mature peptide-coding sequences, exactly duplicated sequence IGHV3-23D, sequences that were treated as one entity in this study. Furthermore, as some germline genes are highly similar, confounding outcomes may occur as a consequence of mutational processes rendering sequences derived from one germline gene more similar to the nucleotide sequence of other germline genes. Such highly similar germline genes (>98% nucleotide identity) were, to avoid erroneous interpretation, not investigated in the present study.

**Figure 14:**
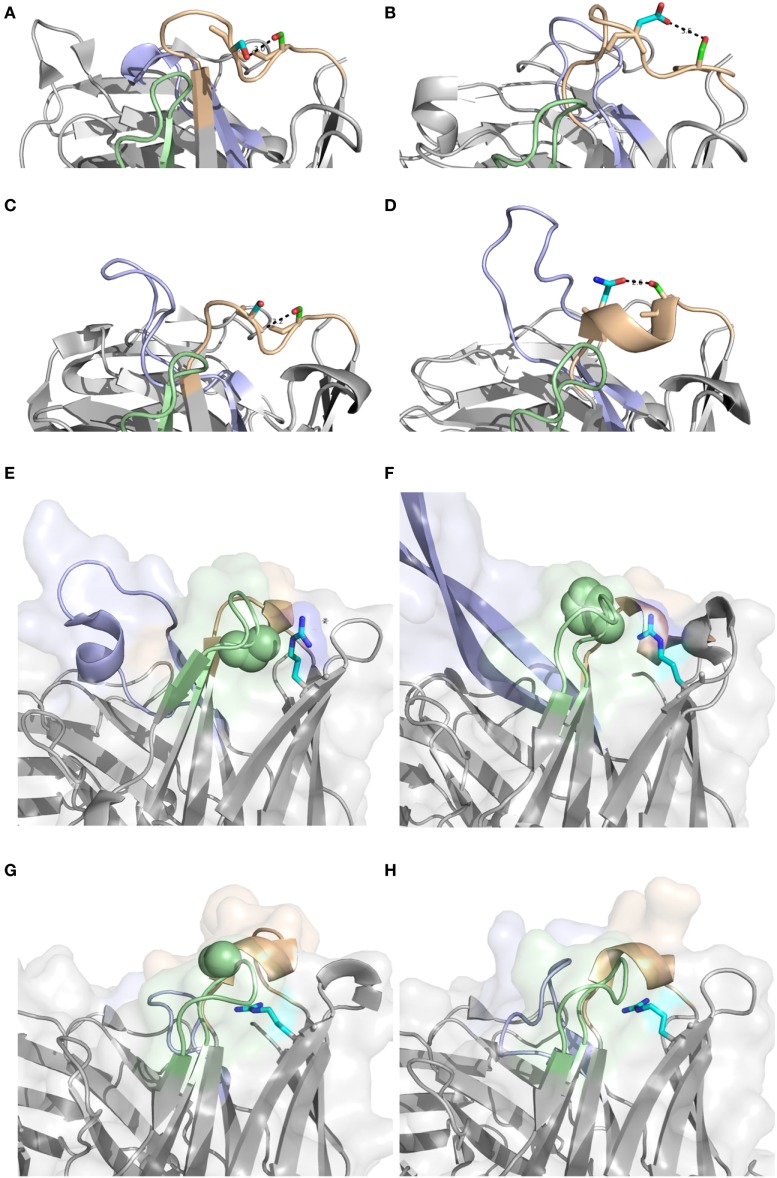
Position 29 of rearranged sequences of different germline gene origins are to different extents targeted by mutagenesis resulting in amino acid substitution (Figure 3). A diversity of potential polar interactions of the side chain of S29 have been implicated such as those to Oγ of S31 (IGHV2-5; PDB: 3QRG) **(A)**, Oδ2 of D31 (IGHV4-39; PDB: 5C6T) **(B)**, backbone O of S31 (IGHV4-59; PDB: 3HI1) **(C)**, and Oδ1 of N36 (IGHV5-51; PDB: 4BUH) **(D)**. H chain CDR1, CDR2, and CDR3, are colored in brown, green, and blue, respectively. Side chain atoms are colored in red (oxygen), blue (nitrogen), green (carbon of residue 29) and cyan (carbon of residue 31 or 36). Position of R80 (carbon of side chain shown in cyan) and residue 58 (residues of the side chain as spheres). Structures PDB: 3×3G (with P58) **(E)** and PDB: 3U1S (with H58) **(F)** both derived from IGHV1-8, and PDB: 3FZU (with S58) **(G)** and PDB: 2R56 (with G58) **(H)**, both derived from the IGHV3 subgroup, are shown. H chain CDR1, CDR2, and CDR3, are colored in brown, green, and blue, respectively.

Antibodies of different germline gene origins differed substantially in terms of diversified residues, in agreement with recent findings (46). There is thus a solid basis for defined, preferred germline-centric paths of antibody evolution. Certainly, driving forces that promote higher substitution frequencies may relate to affinity maturation, stability enhancement etc. It is likely that part of the observed differences relate to the presence or absence of sequences acting as hot-spots for the mutational machinery (47). However, structural analysis have previously demonstrated that amino acids that are in mutational hot-spots are not more likely to actually undergo substitution during somatic hypermutation, suggesting that such hot-spots “are not a major driving force in determining which residues are mutated” (48). In any event, IGHV2-5, IGHV4-39, IGHV4-59, IGHV5-51, and IGHV6-1 all encode S29, a residue capable of different interactions in different antibodies (Figures 14A–D). It is, however, only in sequences derived from IGHV5-51 that this residue is targeted by extensive substitutions. This gene is also the only one among the five that carries a mutational hot-spot motif affecting this codon. If this is a selected, germline-encoded feature preventing extensive, non-functional substitution of products derived from the other four genes, or not, is currently not known. However, if this is not the case, there is a capacity for antibody evolution in antibodies derived from some genes that is not efficiently explored by the human immune system.

Beyond hot-spot involvement in the orchestration of substitution, it is also likely that structural consideration in many cases guide the ability of antibodies of different germline gene origins to tolerate or even prefer substitutions. Certainly, loops belonging to different canonical classes (49, 50), positioning side chains with identical residue numbers in entirely differently orientations and environments, may affect their ability to structurally accept substitutions. Importantly, some germline genes encode unusual residues in some positions, the side chain of which may be suboptimal for its environment. We envisage that such unusual residue may be more commonly mutated even if they reside in FR, often attaining the more common residue following substitution, as illustrated by residues 53 of IGHV3-11 and residue 71 or IGHV1-18. Furthermore, residue 80, a residue in the upper core of the variable domain, with particular importance for the conformation of CDRH2 (38) provide interesting insight into germline-directed paths for antibody evolution. The common diversification of R80 in products with an origin in IGHV1-8 (in contrast to the lack of diversification of R80 in products derived from IGHV3 subgroup genes) (Figures 6 and 9) is not associated with the presence of a mutational hotspot in IGHV1-8 affecting codon 80 (Figure S3 in Supplementary Material). Possibly R80 is less important for maintenance of the integrity of the domain or the general architecture of the binding site in antibodies derived from this germline gene. Interestingly, reorientation of R80 has been demonstrated in one antibody (24) with a likely origin in IGHV1-8. This was also associated with a reorientation of CDRH2 as determined by X-ray crystallography (Figures 14E-H). We hypothesize that substitution of R80 in products originating from genes like IGHV1-8 may be part of an efficient route to evolve antibody functionality while still being tolerated in terms of structural stability. We hypothesize that some germline genes may even have an inherent need, or, if one so-prefer, capacity, for evolution that is not present in other germline genes. In all, the reason for such high substitution frequency *in vivo* may differ between antibodies of different germline gene origins. Future studies will have to address the difficulties encountered by, or alternatively extended opportunity of, the cells producing antibodies derived from these germline genes to gain an advantage in the race for selection through the affinity maturation processes occurring in germinal centers.

Residues beyond CDR may interact with antigen or contribute indirectly to the architecture of the binding site. There are, however, many definitions of CDRs (8–12) apart from the one used in this study (13), definitions that accommodate different viewpoints of what constitutes an antigen binding site. Indeed, substantial diversity is apparently tolerated, or even selected for, in residues in immediate proximity to CDRs as defined by IMGT (Figures 1 and 5). For instance, codon 55 of different germline genes encodes very different side chains. Is the observed difference in mutability solely a result of the presence or absence of a mutational hotspot, or is it also directed by a difference in importance of this residue for establishment of a core antigen-interacting surface (51) in products encoded by the different germline genes? It is furthermore evident that many other residues in FRs, even those belonging to the cores of the antibody fold and to the VH/VL interface, may harbor extensive diversity. We envisage that such diversity, when structurally tolerated may contribute substantially to improved biophysical properties of the encoded antibody or even to affinity maturation, for instance through stabilization of the binding site during affinity maturation (52). In particular, an area with CDR-like potential, CDR4, that resides in a loop adjacent to the classical CDRs in the folded structure have been defined and exploited (11, 39, 48, 53). It has been suggested to be able to accommodate extensive diversity (39). We have now identified that some, but not all, germline genes introduce diversity in this loop. Such diversity may contribute to functional evolution of antibodies of such germline gene origins. In all, the preferred paths of evolution of antibody V domains extend substantially beyond conventional CDRs in ways directed by an antibody’s germline gene origin.

Antibody variable domains, indeed, diversify not only by substitution but also through insertion and deletion of residues into the variable domain sequence (31, 32). In this study, we observed germline gene-inherited patterns that differently target genes with such insertions and deletions, not only in conventional CDRs but also in CDR4 (Figure 12; Figure S5 in Supplementary Material). In the past, we hypothesized that the presence of repetitive codons might be one feature that targets such modifications to a particular part of a gene (54). It is also conceivable that parts of VH domains of different germline gene origins are able to structurally harbor such diversity to different extents, a factor that certainly needs further assessment. Nevertheless, it is conceivable that any immune response that relies on introduction of sequence insertion and/or deletion *in vivo* will only recruit members derived from those germline genes that introduce such diversification with ease in critical parts of the sequence. Important immune responses that require such modification have been reported (5, 55) and other responses relying on such evolution, such as those targeting occluded sites, will likely be described in the future. By understanding the ability of particular germline genes to diversify by insertion and deletion, it will be possible to develop our understanding of selection of germline genes made by the immune system in the generation of these particularly difficult immune responses.

Overall, the diverse pattern of diversification even beyond conventional CDR likely complicates computational efforts to assess the involvement of selection during antibody development. We employed one such analysis technology on highly expressed IgG H chain V domain sequences encoded in BM but found no evidence of a positive selection force in the mutational pattern targeting CDR. Our findings are in line with past studies demonstrating a failure to identify evidence of positive selection in CDR, while evidence of negative selection of modifications in FR is detected (43, 44). In agreement with a recent study (46), and given the diversity of paths through which antibodies of different origins evolve, we suggest that any approach to assess selection ought to take germline gene-specific mutational patterns as found in selected and non-selected repertoires into account and not rely entirely on an analysis of mutations based on current global CDR definitions. Processes to facilitate analysis of selection in a germline gene origin-centric fashion have been initiated elsewhere (46). We foresee that such a development will be required if computational approaches are to accurately address the impact of selection on antibody repertoire development. In some situations, such as IgE responses, this aspect is a matter of substantial biological controversy (56, 57) and certainly need further investigations, the outcomes of which will impact our understanding of fundamental biological processes associated with disease.

In summary, we identified germline gene-unique patterns of evolution that occur during hypermutation of antibodies of diverse IGHV germline gene origins. Our findings extend the findings of a recent study, published during preparation of the present manuscript, that identified gene-specific substitution profiles of antibodies of different germline gene origins (46). Collectively, we have demonstrated a diversity of paths taken by antibodies of different germline gene origins to evolve by somatic hypermutation, including not only base substitution but also processes of codon insertion and deletion. Our study forms the basis for improved understanding of molecular evolution as it proceeds in immune responses *in vivo* and establishes a foundation for future germline gene origin-centered analysis approaches.

## Ethics Statement

This study was carried out in accordance with the recommendations of Regionala etikprövningsnämnden (Lund). All subjects gave written informed consent in accordance with the Declaration of Helsinki. The protocol was approved by the Regionala etikprövningsnämnden (Lund).

## Author Contributions

UK: bioinformatic pipeline development and bioinformatic analysis, manuscript preparation, and approved the final manuscript. HP: conceived the study, manuscript preparation, and approved the final manuscript. FL: initial bioinformatic pipeline development, manuscript preparation, and approved the final manuscript. LG: patient management, manuscript preparation, and approved the final manuscript. MO: conceived the study, bioinformatic analysis, main responsibility for manuscript preparation, and approved the final manuscript.

## Conflict of Interest Statement

The authors declare that the research was conducted in the absence of any commercial or financial relationships that could be construed as a potential conflict of interest.

## Footnotes

^1^http://www.imgt.org.

^2^http://www.rcsb.org/pdb/.

^3^http://selection.med.yale.edu/baseline/.

## Abbreviations

BM, bone marrow; CDR, complementarity determining region; FR, framework region; H, heavy; V, variable; VH, heavy chain variable; VL, light chain variable.
