# Supplementary material for: Antibody Heavy Chain Variable Domains of Different Germline Gene Origins Diversify through Different Paths
Source: Front Immunol. 2017 Nov 13;8:1433. doi: 10.3389/fimmu.2017.01433 (PMC5694033; doi:10.3389/fimmu.2017.01433)
Supplement: Supplementary file 1 [file Data_Sheet_1.DOCX]

# Supplementary information

**Antibodies of Different Germline Gene Origins Diversify through Different Paths**

Ufuk Kirik^1,§^, Helena Persson^2^, Fredrik Levander^1,3^, Lennart Greiff^4,5^, Mats Ohlin^1,6,7 *^

^1^ Dept. of Immunotechnology, Lund University, Lund, Sweden

^2^ Science for Life Laboratory, Drug Discovery and Development Platform & School of Biotechnology, KTH-Royal Institute of Technology, Stockholm, Sweden

^3^ National Bioinformatics Infrastructure Sweden (NBIS), Science for Life Laboratory, Department of Immunotechnology, Lund University, Lund, Sweden

^4^ Dept. of Clinical Sciences, Lund University, Lund, Sweden

^5^ Dept. of Otorhinolaryngology, Head and Neck Cancer, Skåne University Hospital, Lund, Sweden

^6^ Science for Life Laboratory, Drug Discovery and Development Platform, Human Antibody Therapeutics, Lund University, Lund, Sweden

^7^ U-READ, Lund School of Technology, Lund University, Lund, Sweden

^§^ Current affiliation: Disease Systems Biology Program, University of Copenhagen, Copenhagen, Denmark

* Corresponding author: Dept. of Immunotechnology, Lund University, Medicon Village building 406, S-223 81 Lund, Sweden; telephone: +46-462224322; e-mail: mats.ohlin@immun.lth.se

**Supplementary Table 1.** Number of sequence reads representing analysed germline genes from each BM-derived transcriptome that were used in the analysis of substitutions in IGHV-encoded sequence evolution.

| Germline gene | Number of sequences | | | | | |
| --- | --- | --- | --- | --- | --- | --- |
|  | donor 1 | donor 2 | donor 3 | donor 4 | donor 5 | donor 6 |
| IGHV1-8 | 2090 | 7276 | 2513 | 3582 | 6543 | 15241 |
| IGHV1-18 | 8964 | 14727 | 9873 | 5901 | 9491 | 14821 |
| IGHV2-5 | 9131 | * | 7039 | * | 7567 | * |
| IGHV3-7 | 19102 | 15006 | 7815 | 11756 | 9994 | 22336 |
| IGHV3-11 | * | 10784 | * | 4259 | * | 13416 |
| IGHV3-21 | 8751 | 14618 | 9209 | 8992 | 11959 | 15145 |
| IGHV3-23 | 29255 | 34571 | 24607 | 30269 | 36098 | 51949 |
| IGHV4-39 | 16277 | 12049 | 14247 | 26924 | 13492 | 10167 |
| IGHV4-59 | 19195 | 15877 | 15279 | 21392 | 13811 | 29300 |
| IGHV5-51 | 8816 | 12976 | 17066 | 24944 | 12091 | 16640 |
| IGHV6-1 | 2439 | 3589 | 802 | 1656 | 5326 | 4433 |

* Not used for studies of substitutions, as the donor was heterozygous for genes encoding different protein sequences from codon 27-104 in the germline configuration.

**Supplementary Figure 1.** Nucleotide sequence similarity in codons 27-105 between germline genes investigated in this study and other full-length, functional germline genes present in the IMGT database (release 201718-0). Sequence similarities >98% are highlighted in red.


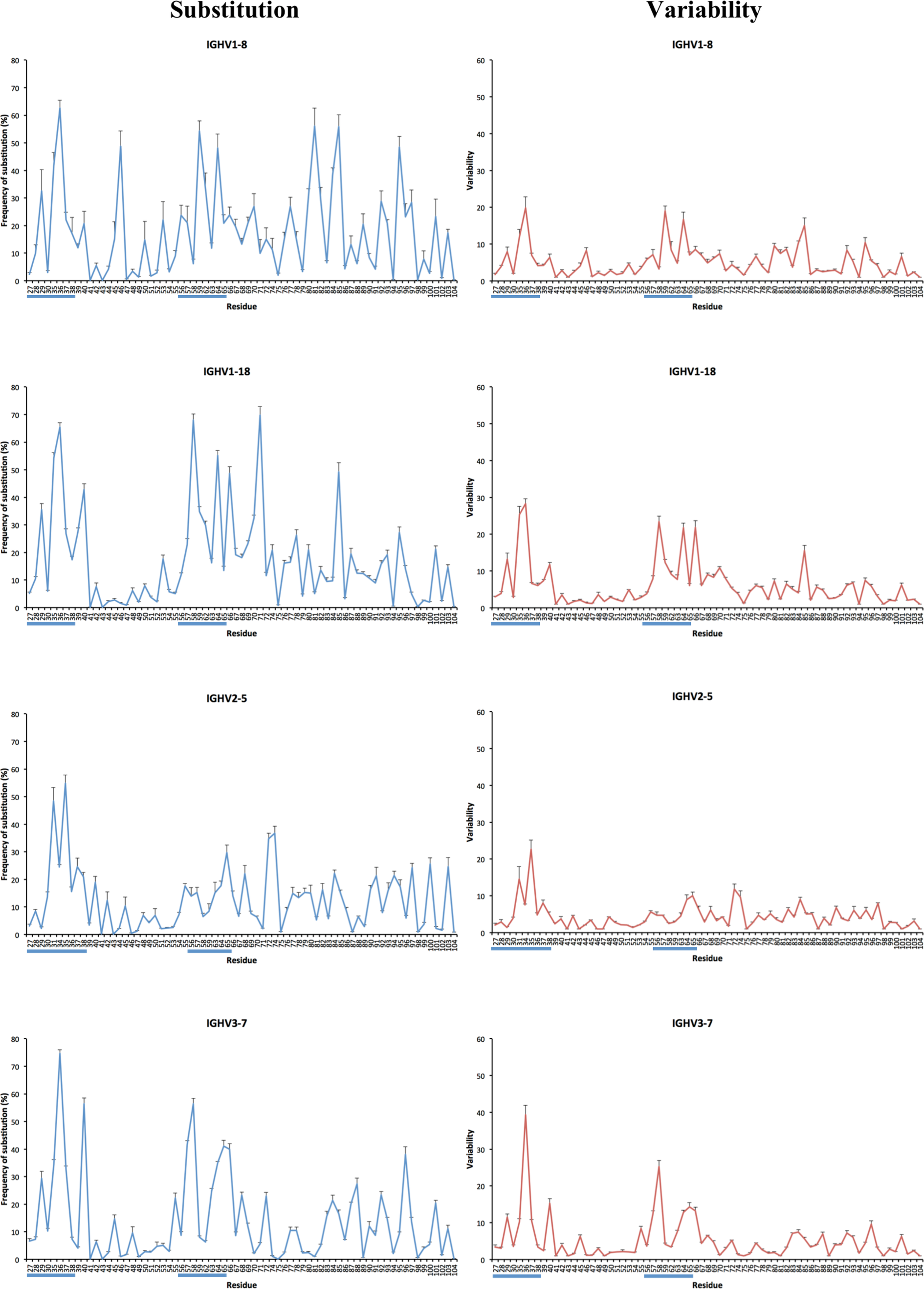


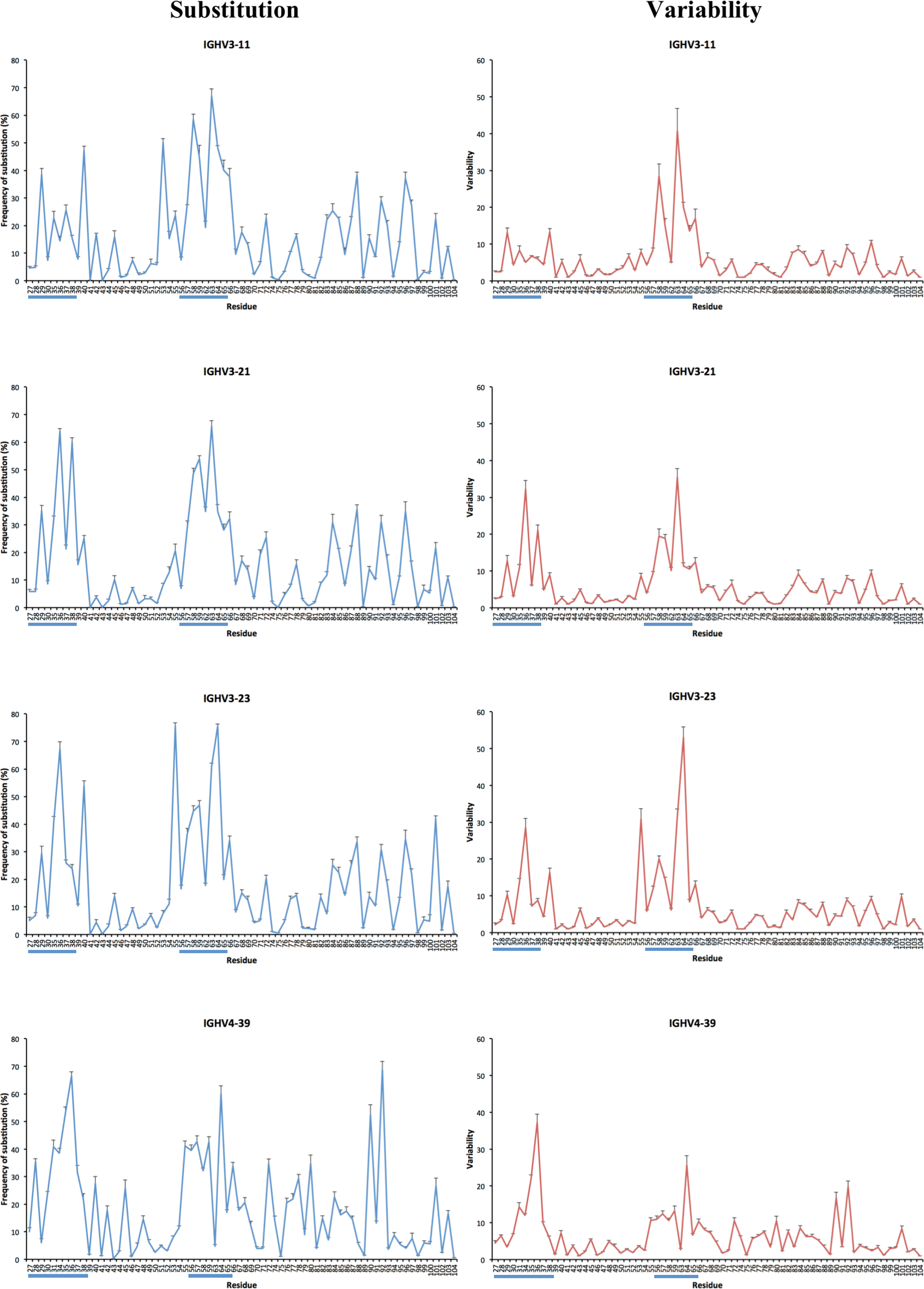


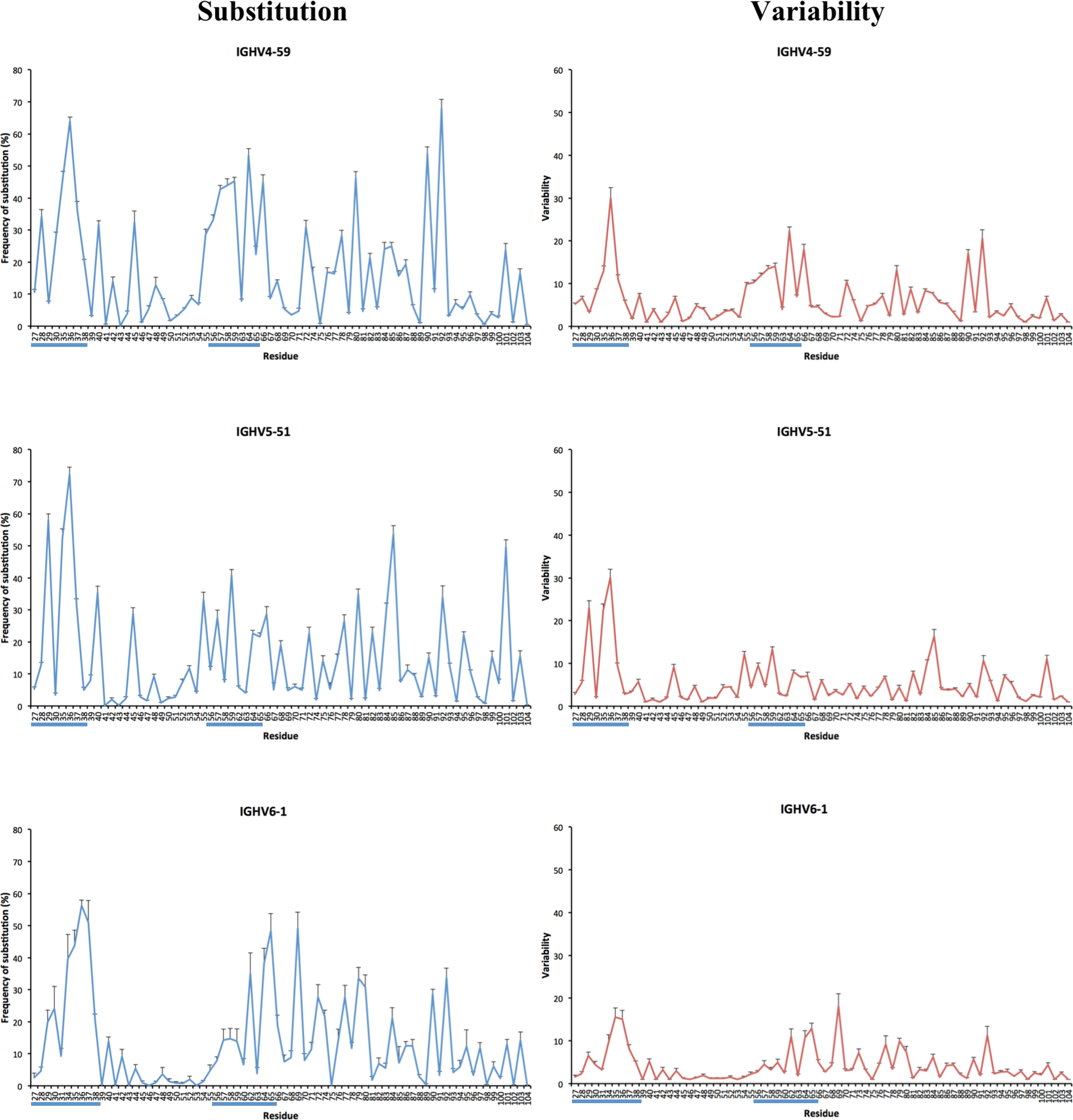


**Supplementary Figure 2.** Frequency and variability of substitutions in transcriptomes encoding VH with an origin in 11 different germline genes. Transcripts derived from genes (IGHV2-5 and IGHV3-11) of donors that had alleles that encoded different protein sequences were not used in this comparison. Variability was calculated as (the number of amino acids encoded by more than 1% of all reads)/(fraction of reads encoding the most common residue). Residues 27-38 code for CDR1 while residues 56-65 code for CDR2 (underlined).

Codon

Gene 23 24 25 26 27 28 29 30 31 34 35 36 37

IGHV2-5*02 tgc acc ttc tct ggg ttc tca ctc agc act agt gga gtg

* † * * *

IGHV4-39*01 tgc act gtc tct ggt ggc tcc atc agc agt agt agt tac

† †* * * *†* *†

IGHV4-59*01 tgc act gtc tct ggt ggc tcc atc ... ... agt agt tac

† †* * * *†

IGHV5-51*01 tgt aag ggt tct gga tac agc ttt ... ... acc agc tac

* * †* ** †† * †† **

IGHV6-1*01 tgt gcc atc tcc ggg gac agt gtc tct agc aac agt gct

† † * †

74 75 76 77 78 79 80 81 82 83 84 85 86

IGHV1-8*01 ggc aga gtc acc atg acc agg aac acc tcc ata agc aca

* ** †

IGHV3-21*01 ggc cga ttc acc atc tcc aga gac aac gcc aag aac tca

* * * *†

IGHV3-23*01 ggc cgg ttc acc atc tcc aga gac aat tcc aag aac acg

† * ** * *

**Supplementary Figure 3.** Location of mutational hotspots (as defined by IMGT V-QUEST) in parts of the sequence of investigated germline genes encoding S29 and R80. Hotspot motifs *WA*/*TW* (*) and *RGYW*/*WRCY* (†), as defined by IMGT V-QUEST, are indicated. Note the presence of mutational hotspots in codon 29 solely in IGHV5-51.


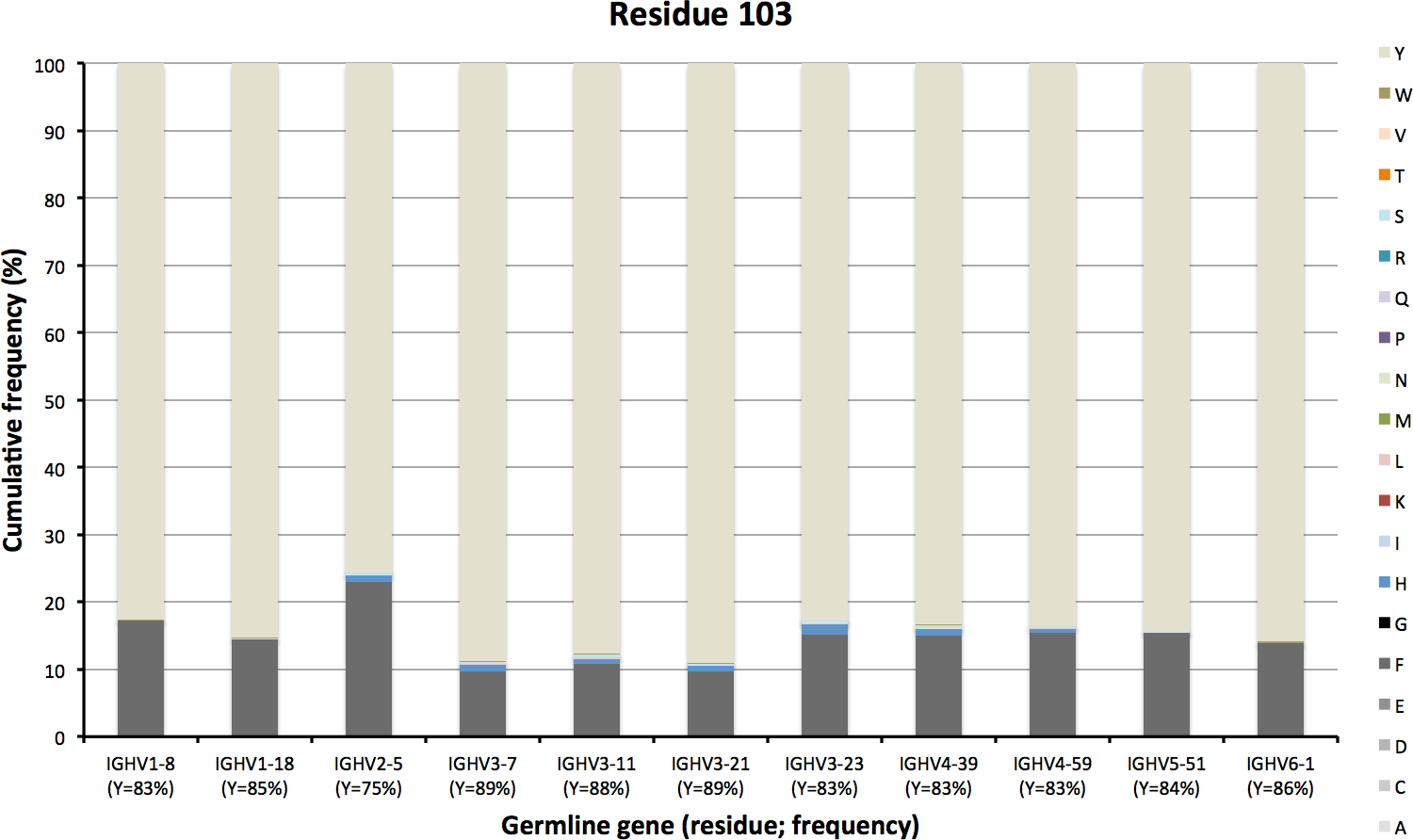


**Supplementary Figure 4.** Substitutions of the VH/VL interphase residue 103 as introduced during somatic hypermutation processes of IgG-encoding genes derived from different germline genes. Substitutions of interphase residues 40 and 42 are shown in Figure 5.


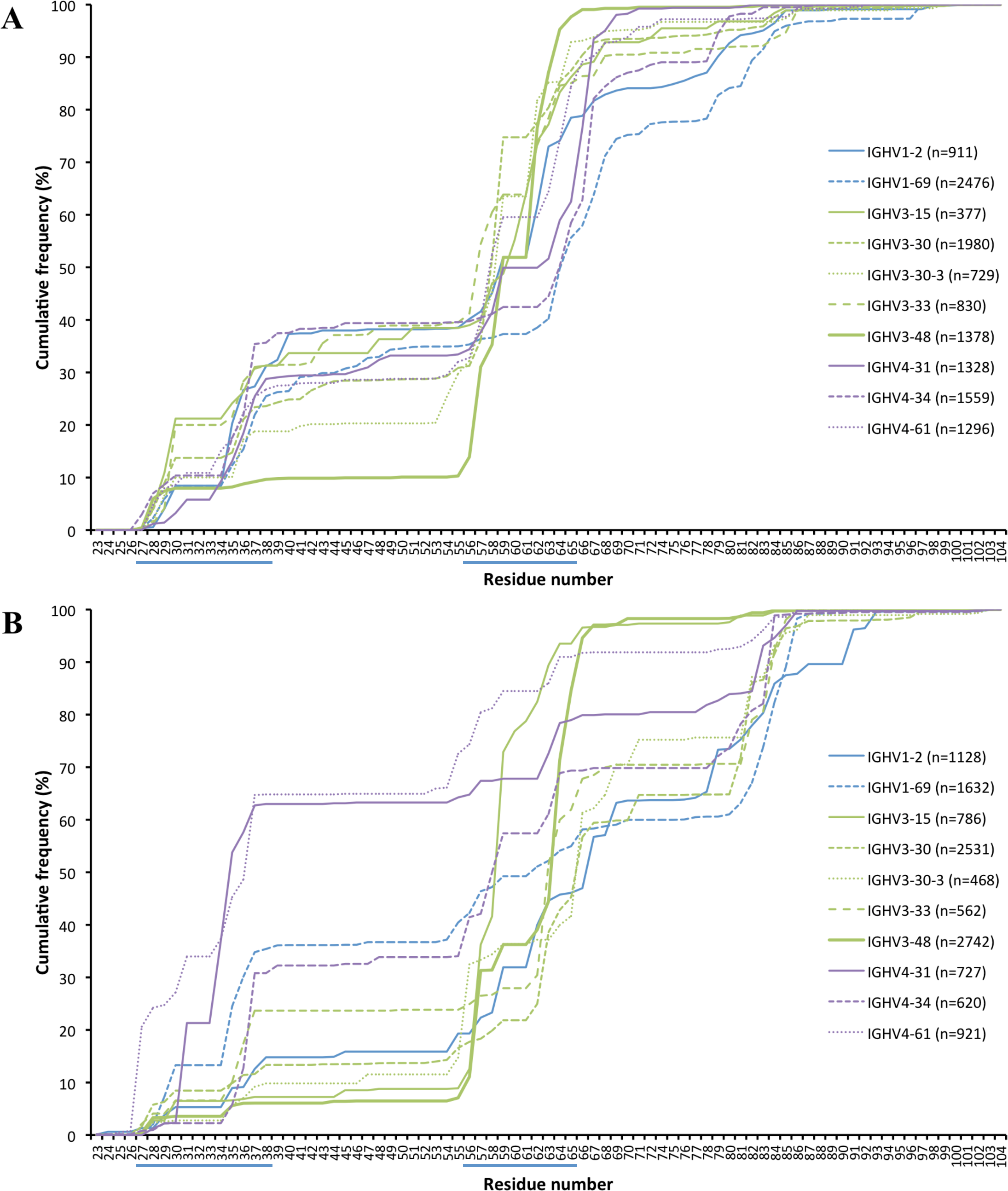


**Supplementary Figure 5.** Cumulative frequency of in-frame codon insertion (A) and deletion (B) (as calculated by IMGT HighV-QUEST) in rearranged genes derived from an extended set of germline genes (irrespective of allele origin). Residues that make up CDR1 and CDR2 are underlined.
